# Weak gene–gene interaction facilitates the evolution of gene expression plasticity

**DOI:** 10.1186/s12915-023-01558-6

**Published:** 2023-03-20

**Authors:** Hao-Chih Kuo, Cheng-Te Yao, Ben-Yang Liao, Meng-Pin Weng, Feng Dong, Yu-Cheng Hsu, Chih-Ming Hung

**Affiliations:** 1 Biodiversity Research Center, Academia Sinica, Taipei, 11529 Taiwan; 2 Division of Zoology, Endemic Species Research Institute, Nantou, 55244 Taiwan; 3 Institute of Population Health Sciences, National Health Research Institutes, Miaoli, 35053 Taiwan; 4 Kunming Institute of Zoology, Chinese Academy of Sciences, Kunming, 650223 Yunnan China; 5 Department of Natural Resources and Environmental Studies, National Dong Hwa University, Hualien, 97401 Taiwan

**Keywords:** Co-expression connectivity, Gene expression plasticity, High-altitude adaptation, Reciprocal transplant experiments, RNA sequencing

## Abstract

**Background:**

Individual organisms may exhibit phenotypic plasticity when they acclimate to different conditions. Such plastic responses may facilitate or constrain the adaptation of their descendant populations to new environments, complicating their evolutionary trajectories beyond the genetic blueprint. Intriguingly, phenotypic plasticity itself can evolve in terms of its direction and magnitude during adaptation. However, we know little about what determines the evolution of phenotypic plasticity, including gene expression plasticity. Recent laboratory-based studies suggest dominance of reversing gene expression plasticity—plastic responses that move the levels of gene expression away from the new optima. Nevertheless, evidence from natural populations is still limited.

**Results:**

Here, we studied gene expression plasticity and its evolution in the montane and lowland populations of an elevationally widespread songbird—the Rufous-capped Babbler (*Cyanoderma ruficeps*)—with reciprocal transplant experiments and transcriptomic analyses; we set common gardens at altitudes close to these populations’ native ranges. We confirmed the prevalence of reversing plasticity in genes associated with altitudinal adaptation. Interestingly, we found a positive relationship between magnitude and degree of evolution in gene expression plasticity, which was pertinent to not only adaptation-associated genes but also the whole transcriptomes from multiple tissues. Furthermore, we revealed that genes with weaker expressional interactions with other genes tended to exhibit stronger plasticity and higher degree of plasticity evolution, which explains the positive magnitude-evolution relationship.

**Conclusions:**

Our experimental evidence demonstrates that species may initiate their adaptation to new habitats with genes exhibiting strong expression plasticity. We also highlight the role of expression interdependence among genes in regulating the magnitude and evolution of expression plasticity. This study illuminates how the evolution of phenotypic plasticity in gene expression facilitates the adaptation of species to challenging environments in nature.

**Supplementary Information:**

The online version contains supplementary material available at 10.1186/s12915-023-01558-6.

## Background

A NASA twins study revealed that an astronaut showed plastic changes in phenotypes, including telomere length and gene expression, in the extreme environment of space compared against his identical twin brother on Earth [1]. In fact, phenotypic plasticity—multiple phenotypes that express from one genotype in response to environmental shifts—is ubiquitous in nature. This biological trait enables individuals to cope with variable environments encountered during their lifespans. However, when an organism colonizes a new environment outside of its regular ecological range, phenotypic plasticity can be beneficial or harmful (or neutral) to the organism because the changed phenotypes may move closer to or farther from new trait optima [2]. Such environmentally induced phenotypic changes may influence the potential of subsequent genetic changes to achieve adaptation [3–7]. Interestingly, over the adaptation process, phenotypic plasticity itself may also be subject to evolution [4, 7–10]. In the past two decades, studies have reported evolutionary changes in phenotypic plasticity between locally adapted populations [11–15].

The evolution of plastic response has become a focus of plasticity research [16–18]. For example, when lowland animals are exposed to high mountains, they produce excessive red blood cells (erythropoiesis) coupled with decreased hemoglobin-oxygen affinity, both of which helps deliver more oxygen at normoxia but does the opposite under environmental hypoxia. These are plastic changes possibly arising from a response to the lack of enough healthy red blood cells to carry adequate oxygen to tissues (anemia) in lowland [19]. By measuring hemoglobin content in hypoxia- and normoxia-acclimated deer mice (*Peromyscus maniculatus*), Lui et al. [20] demonstrated evolutionary changes in the plastic erythropoietic responses between mice native to highland and those native to lowland—the highlander has evolved with a relatively blunted plastic response. In this case, adaptation to high altitude was preceded by the maladaptive ancestral plasticity, which brought the trait (hemoglobin content) away from the local optimum, and then such plasticity itself also altered over the adaptation process. However, it remains uncertain whether traits showing maladaptive plasticity are more likely to have their plastic responses evolved during adaptation to new environment compared with other traits.

Gene expression is a “molecular phenotype” [21] and may show plastic changes in response to environmental differences preceding genetic-based changes [22]. Recent transcriptomic studies have examined how the direction of gene expression plasticity relative to following genetic-based changes influences organisms’ adaptation to new environments [22–26]. Plastic changes in gene expression are classified as “reinforcing” and “reversing” when they are in the same and the opposite directions, respectively, with the adaptive genetic-based changes [23]. These studies found more genes showing reversing plasticity than reinforcing plasticity when organisms face new environments. However, given that most aforementioned studies were based on artificial environmental gradients (with [22] as an exception), the prevalence of reversing over reinforcing plasticity in natural populations requires further research.

More recently, a study demonstrated the prevalence of evolution in gene expression plasticity during adaptation to new environment and, intriguingly, that the derived plasticity often rendered descendant populations similar to ancestral ones in their gene expression levels when moving back to the ancestral environment [27]. However, the factors regulating the evolution of gene expression plasticity remain poorly understood. While it is controversial whether plasticity is a direct target of selection or it evolves as a byproduct of selection on traits carrying different plasticity [27–29], it is likely that both adaptation-associated genes and their regulatory genes are involved in the evolution of plasticity [30]. In addition, the expression variation of genes may be confounded by other genes that are functionally connected to them [31, 32]. Indeed, studies have suggested that gene expression interdependence or protein–protein interaction may impose constraints on expression variation, genetic divergence, or even body plan development due to gene pleiotropy ([32–36]; but see [37]). Thus, epistatic interactions among adaptation-associated genes and other genes may shape plasticity evolution [36, 38], a hypothesis that needs more experimental investigations especially for non-model species. Therefore, we examine whether expression interdependence determines the evolution of gene expression plasticity based on both genes involved in altitudinal adaptation and the whole transcriptome data in a wild bird.

Experimental studies to examine the plastic responses of vertebrates in their natural (or semi-natural) environments are difficult to conduct and are thus rare [39]. Common garden experiments that keep populations from different environments in the same garden can be used to examine the genetic underpinnings of phenotypic differences by controlling environment-induced variation [40]. However, estimating evolutionary change in phenotypic plasticity requires reciprocally transplanting populations native to old and new environments to different gardens resembling their respective environments [11]. Here we examine the gene expression plasticity across altitudes in the Rufous-capped Babbler (*Cyanoderma ruficeps*), a songbird with a wide elevational range (0–3000 m) in the mountainous island of Taiwan. This bird arrived in Taiwan from the Asian mainland during the Early Pliocene (~ 4 Ma) [41], approximately one million years after island emergence [42], when the island was still relatively low and prior to the acceleration of orogeny at 2–3 Ma [43, 44]. Therefore, the bird’s Taiwanese montane populations were presumably derived from its lowland ancestors on the island. By setting common gardens at similar elevations to this bird’s lowland and montane habitats and performing reciprocal transplants, we jointly manipulated multiple ecological factors (e.g., temperature or oxygen pressure) to examine its plastic responses to ecological changes associated with natural colonization and the evolution of its plasticity.

Plastic responses may vary among traits [12], and the variation is evolutionarily important because adaptation to a new environment often involves a combination or series of traits, rather than a single one [5]. In this study, we compared gene expression plasticity patterns between two organs—the brain and the liver—that govern different traits and functions. The liver is regularly exposed to various foreign molecules that enter the body through the gut and plays a critical role in the immune system [45, 46]. Studies have reported divergent copy numbers or selection signals in immune genes across the altitude for several avian species [47, 48], suggesting the importance of immunological function in altitudinal adaptation. In addition, gene expression in the liver of birds, mammals, and fish is known to respond to environmental stress such as temperature or hypoxia stress [17, 49–52]. On the other hand, the brain governs most behavioral traits and also coordinates most activities of the whole body. Such complicated and often more instantaneous requirements warrant the brain to be more flexible than the liver to cope with local, subtle environmental changes or disturbances across altitude. Gene expression in the brain is also found sensitive to hypoxia [53, 54]. Accordingly, we predict that gene expression in the brain and the liver has different altitudinal plasticity patterns and different tendencies of plasticity evolution.

In this study, we aimed to explore the regulatory mechanism of plasticity evolution by examining whether the evolution of gene expression plasticity is determined by plasticity direction relative to following genetic-based changes (reinforcing or reversing) and/or plasticity magnitude. We further tested the role of expression interdependence among genes in the above relationship. We first analyzed the liver and brain transcriptome profiles of transplanted and control groups from lowland and montane populations to identify genes involved in the adaptation of Rufous-capped Babblers to the high attitude in Taiwan. With these genes, we confirmed a dominant role of reversing ancestral plasticity in the bird’s altitudinal adaptation. Importantly, we found that the magnitude of ancestral plasticity greatly determined plasticity evolution. Interestingly, we found such a relationship pertinent to not only adaptation-associated genes but neutral ones as well. We hypothesized that functional/regulatory interdependence caused the observed transcriptome-wide relationship between the magnitude and evolution of gene expression plasticity. Indeed, we gained supportive evidence that genes with less expression interconnection with other genes tended to exhibit higher degree of plasticity evolution. We further noted that the liver-expressed adaptation-associated genes had larger plastic magnitudes and more frequently display evolving plasticity than the brain-expressed ones. Finally, we superimposed this latter finding on those from Ho et al. [27] to discuss its implications in the Rufous-capped Babbler’s long-term survival in the face of rapidly changing environments.

## Results

### The low-altitude ancestry of Rufous-capped Babblers in Taiwan

**Fig. 1:**
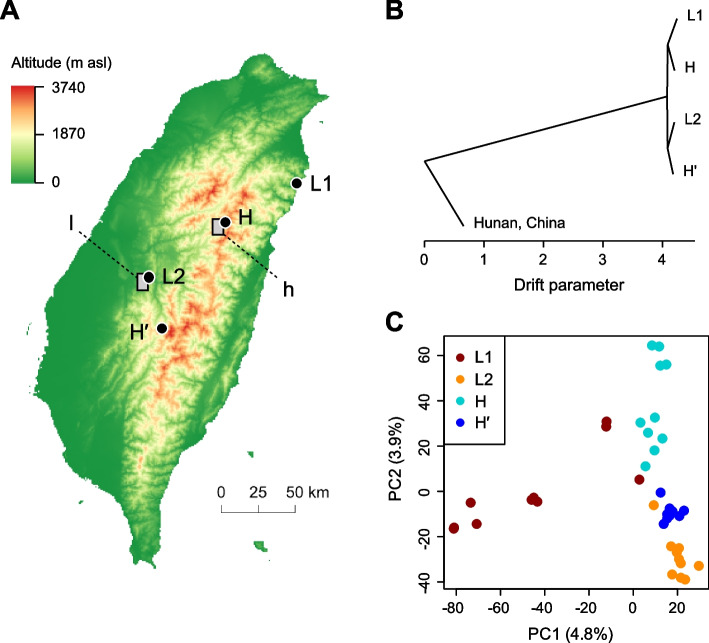
Studied Taiwanese Rufous-capped Babbler populations. **A** A map showing the two low-altitude populations (L1 and L2) and the two high-altitude ones (H and H′). The low- and high-altitude common gardens for reciprocal transplant experiments are also indicated on the map (l and h, respectively). Note that H′ was only used for population genetic analyses, not the transplant experiments. **B** An asymmetric neighbor joining tree for the evolutionary relationships among studied populations plus an additional one from the mainland China. For tree building, a nonreversible model of genetic drift [55] is used to estimate drift-caused changes in allelic frequencies (the drift parameter) between split events; the use of a nonreversible model renders this method being able to root the tree without using outgroups. **C** Principal component analysis (PCA) plot, showing projections of individuals on the first two principal components. Analyses of **B** and **C** were conducted with respective sets of 10,000 randomly selected autosomal single-nucleotide polymorphisms (SNPs) that segregate over the total samples

To infer the direction of gene expression plasticity involved in altitude adaptation, we assumed a lowland origin of Rufous-capped Babblers in Taiwan. To test this assumption, we examined the evolutionary relationships among populations on the island of Taiwan as well as between this island and the Asian continent. We sampled four Taiwanese populations—two from low elevations (L1 and L2 in Fig. 1A) and two from high elevations (H and H′)—and one mainland Chinese population. We generated random sets of 10,000 autosomal single-nucleotide polymorphisms (SNPs) via whole genome resequencing (see “Methods”) and used them to build population trees with a genetic drift model-based method [55]. We obtained consistent population trees across three random SNP sets to have a root between Taiwan and the mainland China (Fig. 1B). This result supported a single origin of the Rufous-capped Babbler in Taiwan, consistent with those of previous studies based on a few genes [41, 56]. Within Taiwan, our population tree revealed two clades, each of which included a high-altitude population and a low-altitude one (Fig. 1B). This tree topology is compatible with the assumed low-altitude ancestry of Rufous-capped Babblers in Taiwan.

Although the four Taiwanese populations were weakly differentiated from one another (*F*_ST_ < 0.05), we applied the principal component analysis to show clusters of individual birds well corresponding to their own populations (Fig. 1C). In addition, we identified distinct genetic groups in an admixture analysis that corresponded to populations L1, H and the combination of L2 and H′, respectively (Additional file 1: Fig. S1). These results confirmed detectable population structure among Taiwanese populations, particularly for the focal populations of our gene expression analyses (L1, H and L2; see below for details).

### Genes that showed expression changes for high-altitude adaptation

**Fig. 2:**
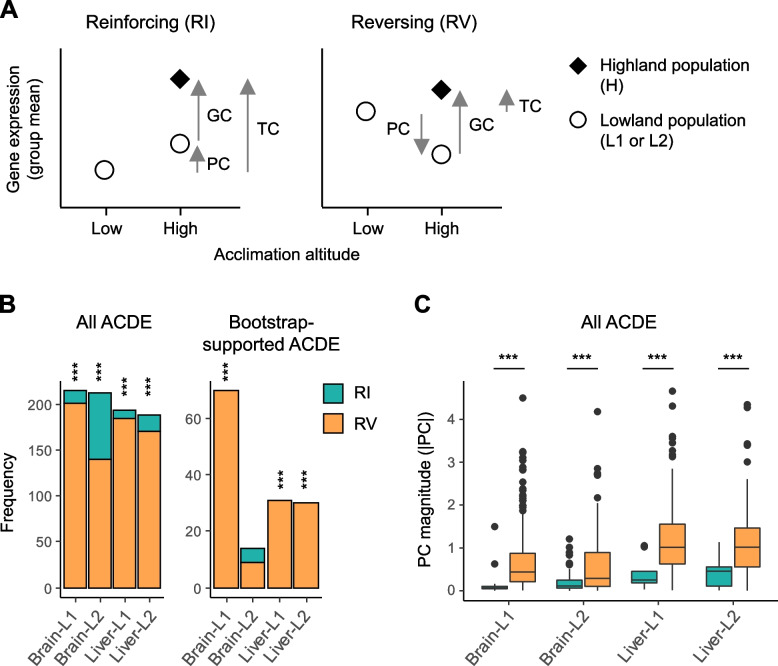
Reinforcing versus reversing expression plasticity (RI and RV, respectively). **A** A schematic showing how to infer RI and RV with samples of the Rufous-capped Babbler populations (coded as in Fig. 1). For genes involved in the bird’s high-altitude adaptation (ACDE genes as defined in the text), we first identified directions of PC and GC expression changes, corresponding to plastic responses of the lowland population and genetic divergence between lowland and highland populations, respectively. We then inferred RI and RV when obtaining the same and opposite PC and GC directions, respectively. Inferences were made for either brain- or liver-expressed ACDE genes, with PC and GC directions identified based on either population L1 or L2 (totally four conditions). The schematic also shows the total expression change between lowland and highland populations in their respective adapted environments (TC), with TC = PC + GC. **B** Frequencies of RI and RV in the ACDE genes and in their subset that acquire strong support in the parametric bootstrap analyses (≥ 950/1000). Unequal RI and RV frequencies are evaluated by two-sided binomial tests (*** indicates *P* < 0.001 while the blank indicates *P* > 0.05. **C** Differences between RI and RV in the absolute PC magnitude (|PC|) that are evaluated by Kruskal–Wallis tests (*** indicates *P* < 0.001)

We then aimed to identify genes associated with altitudinal adaptation of the Rufous-capped Babbler, which likely showed differential gene expression between lowland and montane birds, for downstream analyses on expression plasticity. However, gene expression difference between populations in their own native environments (total expression changes, denoted as TC in Fig. 2A) results from both genetic and environmental difference due to adaptive changes and plastic responses, respectively. To distinguish genetic-based expression changes (denoted as GC in Fig. 2A) from those representing plastic responses (PC), we conducted a common garden experiment. We set a low-altitude common garden (denoted as l in Fig. 1A; 250 m asl) and a high-altitude one (denoted as h; 3000 m asl) at similar altitudes to the lowest and highest habitats of this bird in Taiwan, respectively.

We used RNA sequencing to quantify gene expression in 40 males from three of the four Taiwanese populations used for the population tree reconstruction: 20 from the H population and 10 from each of the L1 and L2 populations. For each population, we equally split the captured birds to the two common gardens for acclimation for a median of 64 days (Additional file 1: Table S1 and Fig. S2). We took 36 whole brain and 39 liver samples from these 40 individuals (Table 1) and used them to obtain 7.2–9.6 (mean = 8.3) million read pairs mapped to 20,559 Babbler genes per brain sample and 10.8–14.8 (mean = 12.4) million to 19,005 Babbler genes per liver sample (Additional file 1: Table S1). We found dramatic differences in expression profiles between tissue types, greatly exceeding those between source populations or acclimating environments (Additional file 1: Fig. S3), and thus, we analyzed brain and liver samples separately. Given that the transplanted birds were adults, we did not examine gene expression that was determined during early development stages, but focused on expression responses after development in this study.

**Table 1:** Sampling of the Rufous-capped Babblers for studying gene expression. N.h and N.l: numbers of individuals acclimated to high- and low-altitude environments, respectively (brain and liver samples presented before and after commas, respectively)

Locality	Code	Altitude (m asl)	N.h	N.l
Meifeng and Dayuling, Nantou County	H	2200–2600	10, 10	10, 10
Nan'ao, Yilan County	L1	< 100	4, 4	4, 5
Jiji, Nantou County	L2	< 300	4, 5	4, 5

We identified 258 brain-expressed and 116 liver-expressed genes whose expressions were affected by the non-identical acclimation durations (see “Methods”) and excluded them from downstream analyses. We then identified genes associated with altitudinal adaptation based on the rationale that they would exhibit substantial genetic-based expression differences when contrasting the high- and low-altitude populations (both H vs. L1 and H vs. L2), but a small difference when contrasting the two low-altitude populations (L1 vs. L2; see “Methods”). We quantified these inter-altitudinal (H vs. L) and intra-altitudinal (L1 vs. L2) expression differences using Xiao et al.’s [57] *π*-value, which evaluates expression differences according to both their magnitudes (|log-fold changes|) and significance levels (*P*-values). Altogether, we calculated ∆*π*_1_ = *π*_(H vs. L1)_ – *π*_(L1 vs. L2)_ and ∆*π*_2_ = *π*_(H vs. L2)_ – *π*_(L1 vs. L2)_ for each gene. We then identified genes that were in the top 5% for both ∆*π*_1_ and ∆*π*_2_. We thus identified 222 genes based on the brain samples, of which 216 (97%) had the same regulation directions (i.e., up- or downregulation) in the two H vs. L contrasts. Similarly, we identified 205 genes based on the liver samples, of which 195 (95%) had the same regulation directions in the two contrasts. The observed proportions of genes showing the same regulation directions in L1 and L2 against H were significantly higher than neutral expectation (two-sided binomial tests against a null proportion of 0.67, *P* < 0.001), implying deterministic, rather than neutral, processes in creating the expression differences. We retained genes showing the same regulation directions in the two H vs. L contrasts for downstream analyses and referred to them as ACDE genes for their “altitudinally concordant differential expression”.

We tested for enrichments of the ACDE genes in Gene Ontology (GO) and Kyoto Encyclopedia of Genes and Genomes (KEGG) terms (detailed in “Methods”). No GO or KEGG term was significantly enriched in the brain-expressed ACDE genes after multiple testing corrections (one-sided Fisher exact tests, adjusted *P* > 0.05). In contrast, the liver-expressed ACDE genes were enriched in four Biological Process GO terms alongside two KEGG terms (adjusted *P* < 0.05), all of which were related to immunological function and pathogen defense (Additional file 1: Table S2).

### High-altitude adaptations were largely associated with reversing plasticity in ancestral, low-altitude populations

To understand how gene expression plasticity in the ancestral, lowland populations of the Rufous-capped Babbler influenced their high-altitude adaptation, we examined the plastic responses of ACDE genes in the two extant lowland populations L1 and L2 (denoted as PC in Fig. 2A). Specifically, we aimed to know whether the expression changes to achieve high-altitude adaptation (GC) were more often preceded with plastic responses (PC) in the same or opposite directions—referred to as “reinforcing” and “reversing” plasticity, respectively (Fig. 2A) [23]. For this, we identified genes’ PC directions (up- or downregulated) by comparing a low-altitude population’s individuals that were acclimated to the high- versus low-altitude environments. We then identified genes’ GC directions in the H vs. L contrast (individuals acclimated in the high-altitude environment) and compared their GC and PC directions to infer reinforcing or reversing plasticity. Inferences were conducted separately for brain- and liver-expressed ACDE genes and for L1 and L2 populations (denoted as brain-L1, brain-L2, liver-L1, and liver-L2 conditions). We found significantly higher frequencies of reversing (66–95%) than reinforcing plasticity in all four cases (two-sided binomial tests, *P* < 0.001; Fig. 2B).

To confirm the robustness of unequal frequencies between reinforcing and reversing plasticity with respect to random sampling errors [58], we carried out a parametric bootstrap procedure modified from Ho and Zhang [24] (see “Methods”). This bootstrap approach aimed to identify genes with PC ≠ 0 and GC ≠ 0 resulting from genuine differences rather than random sampling errors. We finally identified 70 and 14 strongly supported (≥ 950/1000 bootstrap replicates) ACDE genes for the brain-L1 and brain-L2 conditions, respectively. Among these, 100 and 64% exhibited reversing plasticity. Similarly, 31 and 30 strongly supported ACDE genes were identified for the liver-L1 and liver-L2 conditions, respectively, both of which showed 100% reversing plasticity. Of the above four cases, genes exhibiting reversing plasticity significantly outnumbered those exhibiting reinforcing plasticity (two-sided binomial test, *P* < 0.001), except the brain-L2 condition (*P* > 0.05; Fig. 2B). Collectively, the results suggest that high-altitude adaptation of Rufous-capped Babblers in Taiwan was largely associated with reversing plasticity in ancestral, low-altitude populations.

In addition, we noticed that ACDE genes exhibiting reinforcing plasticity were characterized with small magnitudes of PC (denoted as |PC|). Indeed, reinforcing plasticity was found to be associated with significantly smaller |PC| compared to reversing plasticity in all four experimental conditions—brain-L1, brain-L2, liver-L1, and liver-L2 (Kruskal–Wallis tests, *P* < 0.001; Fig. 2C). We deem a geometrical cause for the observed pattern: (1) ACDE genes were identified due to their large |GC| and (2) GC and PC contributed to the total change (TC) in the same direction for reinforcing plasticity (visualized in Fig. 2A)—the two factors together led to compressed |PC| in ACDE genes with reinforcing plasticity.

### Frequent evolution of plasticity over the course of adaptation

**Fig. 3:**
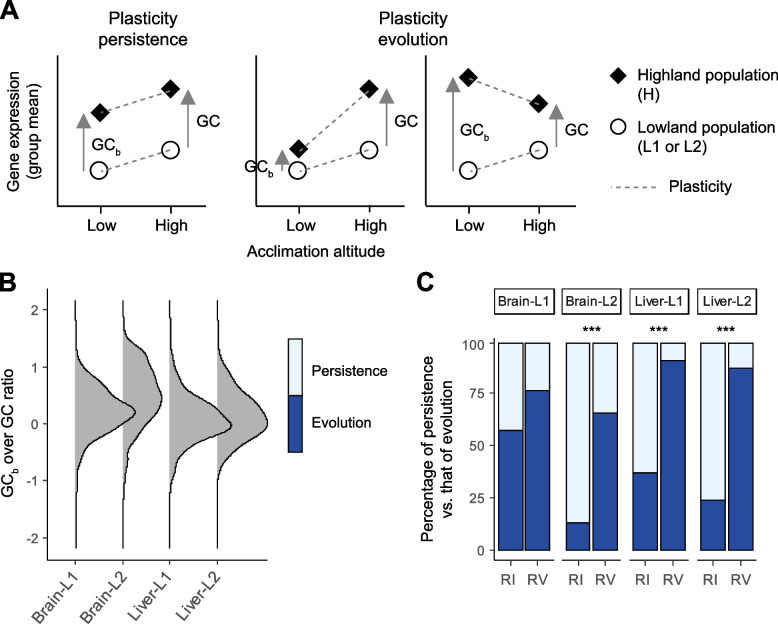
Persistence versus evolution of expression plasticity over the course of adaptation. **A** A schematic for distinction between plasticity persistence and plasticity evolution. Briefly, with two measures—GC and GC_b_—obtained from the studied Rufous-capped Babbler populations, plasticity persistence, and plasticity evolution refer to situations where, respectively, the two measures are about equal to and substantially different from each other. **B** After empirically examining the distributions of the GC_b_-to-GC ratios in our cases (brain- or liver-expressed ACDE genes, two measures derived based on either population L1 or L2), we defined plasticity persistence and plasticity evolution to be with ranges of the ratio as 1 ± 0.5 and 0 ± 0.5, respectively. **C** Percentages of ACDE genes that each exhibits either reinforcing or reversing expression plasticity (RI and RV, respectively) undergo plasticity persistence and plasticity evolution. Varied proportions of plasticity evolution between categories (RI vs. RV) are evaluated by two-sided Fisher exact tests (*** and blanks indicate *P* < 0.001 and > 0.05, respectively)

The impact of phenotypic plasticity on adaptive evolution could be more complex if phenotypic plasticity itself has also evolved during adaptation—a hypothesis requires more empirical evidence. Recent conceptual models distinguish adaptation processes with ancestral plasticity persisting in descendant populations from those with evolved plasticity [7, 16]. Plasticity persistence is characterized with a constant phenotypic difference between ancestral and descendant populations across environments, while plasticity evolution is characterized with non-constant inter-population differences (visualized in Fig. 3A). To examine which model each of the ACDE genes fit, we first measured the expression changes of high- versus low-altitude individuals acclimated to the low-altitude environment (denoted as GC_b_ with “b” for “back” to this ancestral environment). We then determined whether each ACDE gene fit better to the model of plasticity persistence (GC_b_ = GC, Fig. 3A left panel) or plasticity evolution (|GC_b_| <|GC|, Fig. 3A, middle panel). Another possible situation for plasticity evolution is |GC_b_| >|GC| (Fig. 3A, right panel). Nevertheless, we expected scarce of |GC_b_| >|GC| cases because physiological constraints or homeostatic regulation may confine expression variation [7]. Thus, we focused on the cases with |GC_b_| <|GC| to test the plasticity evolution hypothesis.

We categorized genes as having persisting plasticity when GC_b_ and GC were in the same direction and GC_b_ was 1 ± 0.5 GC, and genes as having evolved plasticity when GC_b_ was 0 ± 0.5 GC. Genes showing any other relationships between GC_b_ and GC (i.e., GC_b_ <  − 0.5 or > 1.5 GC) were left unclassified. We chose GC_b_ = 0 as a reference point of plasticity evolution because the distributions of the GC_b_-to-GC ratios had a modal value close to zero in three of the four experimental conditions (Fig. 3B). In the four conditions, 4 − 6% genes were unclassified, fitting with our expectation that the cases with |GC_b_| >|GC| were rare. With these unclassified genes excluded, three of the four conditions showed significantly more genes with evolved plasticity than with persisting plasticity (75 − 89% with evolved plasticity; two-sided binomial tests, *P* < 0.001) while the brain-L2 condition showed no significant difference between the two models (47% with evolved plasticity; *P* > 0.05). The proportion of genes with evolved plasticity further rose to 69 − 100% when we limited analyses to the ACDE genes with strong bootstrap support: significantly unequal numbers of evolved vs. persisting plasticity were found in all (two-sided binomial tests, *P* < 0.001) but the brain-L2 condition (*P* > 0.05). To sum up, genes associated with high-altitude adaptation mostly underwent plasticity evolution (i.e., |GC_b_| <|GC|) such that ancestral and descendant populations had similar gene expression levels in the ancestral (low-altitude) environment (Fig. 3A, middle panel).

### Genes with higher response to environmental change showed higher propensity towards plasticity evolution

Factors that determine the evolution of gene expression plasticity have rarely been examined. We noted that ACDE genes exhibiting reversing plasticity underwent plasticity evolution more frequently than did those exhibiting reinforcing plasticity. This pattern was found in all four experimental conditions and gained statistical support in three of them (two-sided Fisher exact tests, *P* > 0.05 in the brain-L1 condition, otherwise *P* < 0.001; Fig. 3C). To confirm the positive relationship between reversing plasticity and plasticity evolution, we adopted finer categorizations of genes according to degree of evolution in their plasticity. Specifically, we adopted two categorization schemes where three and four bins were set respectively along the spectrum of the GC_b_-to-GC ratio from values ~ 1 (low degree of plasticity evolution) to ~ 0 (high degree of plasticity evolution) (Additional file 1: Fig. S4A; see “Methods”). Under both categorization schemes, we found that the proportions of genes exhibiting reversing plasticity increased with increasing degree of plasticity evolution; the inter-category differences in the reversing plasticity proportions were significant in three of the four conditions (Fisher exact tests, *P* > 0.05 in the brain-L1 condition, otherwise *P* < 0.01; Additional file 1: Fig. S4B). To further confirmed the robustness of the discovered pattern against the arbitrariness of bin settings, we also employed a continuous measure of plasticity evolution: the magnitude of divergence between the lowland and montane populations in their reaction norms (= the interaction between population and acclimation effects in a linear model, visualized in Additional file 1: Fig. S5A; see “Methods”). This continuous measure also showed significantly higher levels of plasticity evolution associated with reversing than reinforcing plasticity in most conditions (Kruskal–Wallis tests, *P* > 0.05 in the brain-L1 condition, otherwise *P* < 0.05; Additional file 1: Fig. S5B).

Why was plasticity evolution positively associated with reversing plasticity? One could argue that the pattern represented an artifact derived from random sampling errors described previously [58]. If so, then we would expect such artifacts more likely to occur in genes without bootstrap support, rendering proportionally more plasticity evolution in this group than in the bootstrap-supported group. However, all four conditions showed trends opposite to the expectation, which was even significant in two of these conditions (Additional file 1: Fig. S6). In other words, plasticity evolution was enriched in bootstrap-supported genes than in genes without bootstrap support, despite the potential artificial inflation in the latter group. Thus, the association between reversing plasticity and plasticity evolution represents a real pattern, with the underlying mechanism requiring in-depth examinations.

**Fig. 4:**
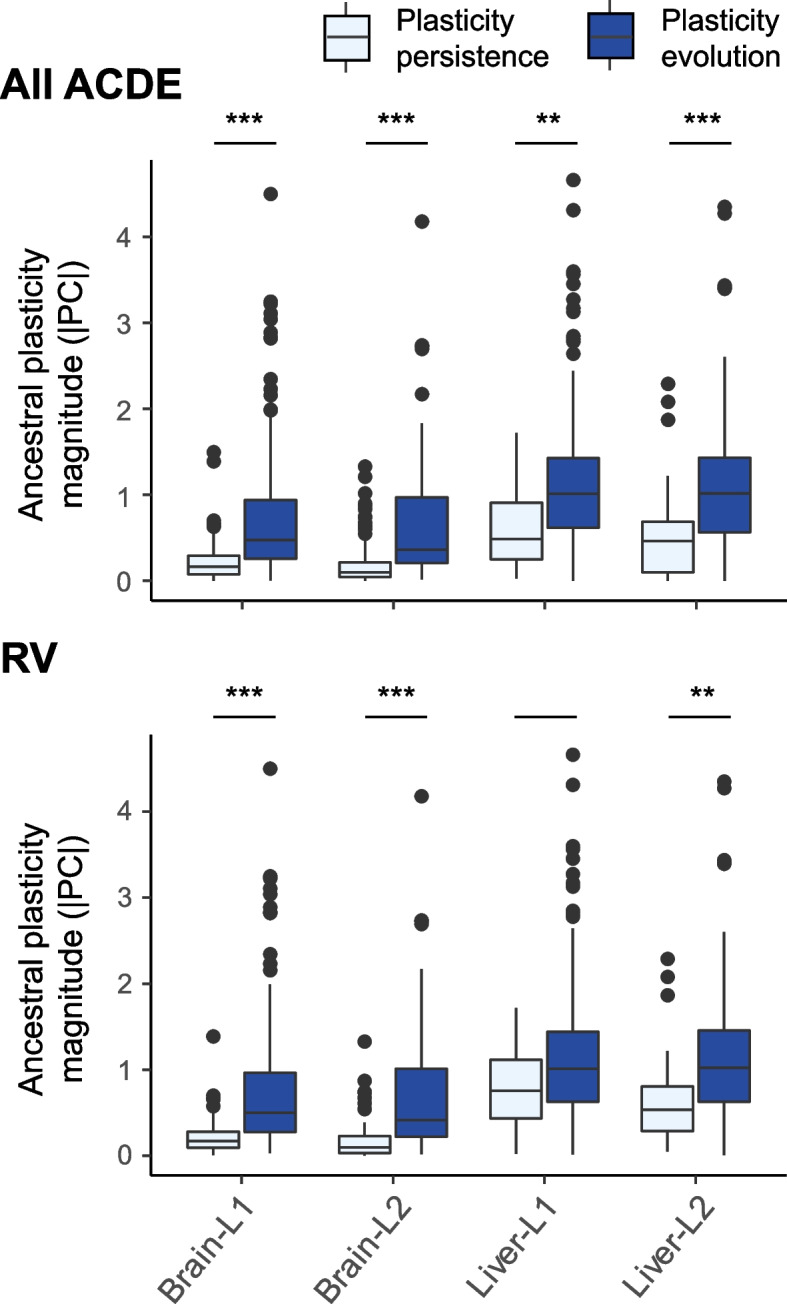
Differences in the ancestral plasticity magnitude (|PC|) between genes showing persisting and evolved plasticity. Comparisons are made for all ACDE genes (upper panel) and for the subset exhibiting reversing ancestral plasticity (lower panel), derived with either brain or liver samples in either H vs. L1 or H vs. L2 population contrasts. Statistical significance is evaluated by Kruskal–Wallis tests (***, ** and blanks indicate *P* < 0.001, < 0.01, and > 0.05, respectively)

Recall that a notable difference between reinforcing and reversing plasticity is the smaller plasticity magnitude (|PC|) in the former type (Fig. 2C). This rendered us to hypothesize that genes whose expressions are less responsive to environmental shifts (i.e., with smaller |PC|) are less likely to undergo plasticity evolution. Supporting our hypothesis, we found significantly larger |PC| in ACDE genes with evolved plasticity than in those with persisting plasticity in all four conditions (Kruskal–Wallis tests, *P* < 0.01; Fig. 4A). Moreover, the same trend was also obtained when limiting the comparisons to either reversing or reinforcing plasticity genes, with reversing-based trends gaining statistical support in three of the four conditions (Kruskal–Wallis tests, *P* > 0.05 in the liver-L1 condition, otherwise *P* < 0.01; Fig. 4B); all four reinforcing-based trends were insignificant (*P* > 0.05; Additional file 1: Fig. S7) supposedly due to lack of statistical power caused by small sample sizes of reinforcing plasticity genes. These findings suggest that the magnitude of the ancestral plasticity determined whether the plasticity subsequently evolved. We also examined the associations between |PC| and plasticity evolution when categorizing genes’ plasticity evolution into either three or four bins as well as when measuring plasticity evolution on a continuous scale as described above. Furthermore, we used the continuous plasticity evolution measure to examine the above association in the bootstrap-supported ACDE genes (counterpart analyses based on binned plasticity evolution could not be performed due to lacks of bootstrap-supported genes in one or more bins). These latter examinations indicated that genes with larger magnitude of plasticity (expression difference between environments) indeed underwent greater degree of plasticity evolution (evolutionary change in plasticity between populations; Additional file 1: Fig. S8, Fig. S9 and Fig. S10) and that this association was robust against random sampling error (Additional file 1: Fig. S10).

We then examined whether the above hypothesis held in the rest of the Rufous-capped Babbler’s genes (i.e., genes that had evolved neutrally or were irrelevant to the altitudinal adaptation) as in the ACDE genes (see “Methods”). With genes’ plasticity evolution either categorized into two, three, or four levels or measured continuously as in the ACDE genes, we found the same positive relationship between |PC| and plasticity evolution in both the brain- and liver-expressed non-ACDE genes (Additional file 1: Fig. S11 and Fig. S12). Therefore, the positive relationship between magnitude and evolution of plasticity was not limited to adaptation-associated genes, but a transcriptome-wide pattern.

### Expression interdependence restricted evolution of expression plasticity

The expression of individual genes often interacts with those of other genes due to functional or regulatory interdependence, constituting co-expression networks [59, 60], in which genes with stronger interconnection have lower evolutionary rates [33, 35]. Thus, we hypothesized that functional/regulatory interconnection among genes may restrict the evolution of their expression plasticity. This hypothesis predicted a negative relationship between gene expression connectivity and the evolution of expression plasticity.

**Fig. 5:**
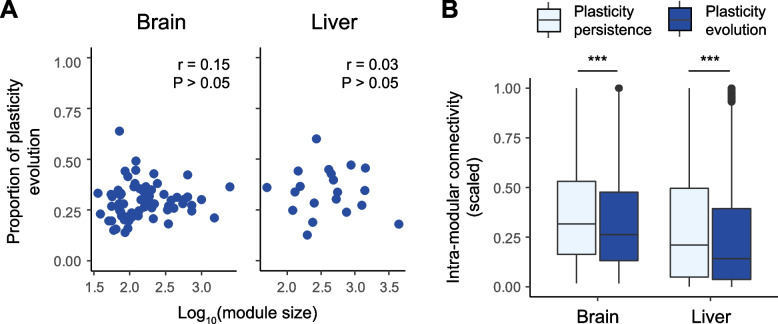
Examining on the negative relationship between plasticity evolution and gene–gene interactions. Here we focus on genes of the whole transcriptomes, with plasticity persistence and plasticity evolution inferred as in Fig. 3B. We applied the WGCNA analyses [61] to the pooled samples of the two extant lowland populations acclimated to either low- or high-altitude common gardens, so to identify co-expression modules. **A** To examine whether plasticity evolution is associated with fewer gene–gene interactions compared to plasticity persistence, we evaluate the Spearman correlation between the module size and the proportions of genes with plasticity evolution in the modules. **B** To examine whether plasticity evolution is associated with weaker interactions than is plasticity persistence, we evaluate their difference in the intra-modular connectivity (*k*_IM_; see the text for details) using the Kruskal–Wallis test (*** indicates *P* < 0.001). Compared to genes with persisting expression plasticity, those with evolved plasticity (**A**) interact with similar numbers of other genes while (**B**) showing weaker gene–gene interactions in the ancestral population

To test the above expectation, we used the weighted gene co-expression network analysis (WGCNA) [61] to identify co-expression modules in the low-altitude populations when they encountered altitudinal change. We obtained 67 and 20 co-expression modules from brain- and liver-based WGCNA analyses, respectively (Additional file 1: Fig. S13). We first examined whether genes with evolved plasticity tended to occur in smaller modules, which showed connection with fewer genes, compared to those with persisting plasticity. Nevertheless, we detected insignificant tendency of occurrence with respect to the module size in both brain- and liver-expressed genes (one-sided Spearman correlation tests, *P* > 0.05; Fig. 5A).

However, genes in the same modules vary in the strength of their interactions with one another. To take this into account, we obtained each gene’s intra-modular connectivity (*k*_IM_, see “Methods” for its definition) [62], which measured the effective level of connections; we further explored the effect of scaling *k*_IM_ by its maximum value per module, which made *k*_IM_ independent from the module size. We found that genes with evolved plasticity had significantly smaller *k*_IM_ than did genes with persisting plasticity regardless of whether scaled or unscaled *k*_IM_ was used (Kruskal–Wallis tests, *P* < 0.001; Fig. 5B, Additional file 1: Fig. S14). We also found a generally consistent pattern across the brain and liver samples that *k*_IM_ decreased with increasing degrees of plasticity evolution when categorizing plasticity evolution into three or four discrete levels (Kruskal–Wallis tests, *P* < 0.001; Additional file 1: Fig. S14 & S15). The same pattern was recovered when plasticity evolution was measured continuously (one-sided Spearman correlation tests, *P* < 0.001; Additional file 1: Fig. S16). Together, we revealed that genes with evolved and persisting plasticity interacted with similar numbers of other genes but differed in the interaction strength—gene with weaker interactions showed greater plasticity evolution.

### ACDE genes had weaker expression interdependence and greater levels of plasticity evolution than other genes

We acquired a consistent pattern across the brain and liver samples that ACDE genes had smaller *k*_IM_ than did non-ACDE genes (Additional file 1: Fig. S17), indicating peripheral positions of the ACDE genes in the co-expression networks. Such differences between ACDE and non-ACDE genes reached statistical significance for both scaled and unscaled *k*_IM_ except the brain-based scaled *k*_IM_ comparison (Kruskal–Wallis tests, *P* > 0.05 in this exceptional case, otherwise *P* < 0.01; Additional file 1: Fig. S17). We further found consistent patterns across the brain and liver samples that ACDE showed significantly larger |PC| and underwent significantly greater levels of plasticity evolution than non-ACDE genes (Kruskal–Wallis tests, *P* < 0.001; Additional file 1: Fig. S18).

## Discussion

In this study, we demonstrated that altitudinal adaptation of a wild songbird—the Rufous-capped Babbler—is predominately associated with genes exhibiting reversing ancestral plasticity. Similarly, recent transcriptomic studies have revealed the dominance of genes with reversing plasticity in adaptations of various taxa to new environments [22–26, 63]. However, the cause of such a pattern is contentious. One study argues that genes exhibiting reinforcing ancestral plasticity adapt to new environments slowly because—with the phenotypes moved towards the optima in new environments by plasticity—they are under weak selection [22]. In contrast, Ho and Zhang [23] argue that genes with reinforcing plasticity are necessarily scarcer than genes with reversing plasticity once a fraction of genes show |PC| >|TC|, with TC (total change) representing the difference in gene expression between populations acclimated to their respective native environments. Given the formula TC = PC + GC (visualized in Fig. 2A), all genes fulfilling |PC| >|TC| necessarily exhibit reversing plasticity, while half of those fulfilling |PC| <|TC| are also expected to exhibit reversing plasticity. This leads to a minimum proportion of genes with reversing plasticity as 0.5, which increases as the proportions of |PC| >|TC| increase. The proportions of |PC| >|TC| increase when genes have larger |PC| and/or smaller |TC|.

The magnitudes of PC may vary among different genes or traits. For example, immune function genes in both humans and mice have been shown to exhibit larger |PC| than the other genes [64]. In our study, 25–53% brain-expressed ACDE and 61–65% liver-expressed ACDE genes showed |PC| >|TC|, which may explain why the proportions of reversing plasticity are higher in the liver-expressed ACDE genes than in the brain-expressed ones (Fig. 2B). The enrichment of immune function genes in the liver-expressed ACDE suggests that the high proportions of |PC| >|TC| in these genes are mainly caused by large |PC|, although the contribution from small |TC| [4] should be scrutinized further.

The liver has to maintain a balanced immune response to protect the body from pathogens and not damage the body by overreacting [46, 65]. This balanced immune response relies on interactions between different immune cell populations [45], which could be tilted when organisms encounter environments outside of adapted altitudinal ranges. Indeed, studies on lowland-adapted species have revealed that their immune cell compositions change when transferred to high-altitude environments [66]. Furthermore, one study found that multiple immune genes in the mouse liver showed plasticity after acclimating to hypoxic conditions, typical of that found at high elevations [49]. Genetic-based modulations in immune gene expression might then rebuild immunity balance in the liver as an organism adapts to new altitudes. In addition, immune genes could diverge across the altitude due to evolutionary responses to various pathogen constitutions. For example, high- and low-altitude populations of the Hawaiian honeycreeper (*Chlorodrepanis virens*) have diverged in genes governing immune responses to avian malaria, which only occurs in the lowlands [67]. Our results, together with those from other avian species showing differentiated immune genes across the altitude [47, 48], suggest that immune gene evolution that is strongly associated with the liver function contributes to altitudinal adaptations in birds.

We showed that expression plasticity in the majority of ACDE genes underwent evolution instead of persisting over adaptation, largely consistent with recent transcriptomic studies [16; but see 26]. We then showed that genes with larger |PC| in the ancestral populations tended to have greater degree of plasticity evolution during adaptation. Ghalambor et al. [22] studied expressions of genes responsible for the adaptation of Trinidadian Guppies (*Poecilia reticulata*) to predator-free environments, and a positive relationship between |PC| in the ancestral population and evolutionary change in plasticity during adaptation was implied in their results. They attributed this pattern to strong selection against a large magnitude of maladaptive plasticity. However, we showed that such a positive relationship between |PC| and plasticity evolution is more likely a general pattern relevant to all genes than a pattern limited to genes under selection (ACDE genes) although the latter tended to have both larger |PC| and greater plasticity evolution.

Consequently, we raised an alternative hypothesis for the observed positive relationship between the magnitude and evolution of plasticity: both |PC| and plasticity evolution of a gene are negatively regulated by the strength of functional/regulatory interactions with other genes. Consistent with our hypothesis, we demonstrated that genes that exhibited higher connectivity to the other genes in co-expression modules evolved less in their expression plasticity compared to those exhibiting lower connectivity. It is worth noticing that ACDE genes showed lower expression connectivity and higher levels of plasticity evolution than other genes. The findings imply that genes subject to lower expression interdependence are more likely to become ones contributing to or associated with adaptation, such as ACDE genes in the case.

The hypothesized relationship between plasticity magnitude and the level of interactions among genes is supported by Papakostas et al*.* [36], which studied protein-expression plasticity in the European Grayling (*Thymallus thymallus*) across a temperature gradient. The fish showed the magnitude of protein-expression plasticity negatively associated with the level of protein–protein interactions. In agreement with this, it has been found that genes that represent network hubs (i.e., with abundant connections to other genes) generally confer phenotypic stability against environmental perturbations [68, 69]. In addition, other recent studies showed that hub genes of transcriptional or protein–protein interaction networks have their *cis-*regulatory regions subject to strong purifying selection that depleted genetic variants in these regions compared to those of peripheral genes [32, 70]. It is likely that such depletions of standing genetic variation in the *cis-*regulatory regions render the hub genes reduced plasticity evolution.

Some caveats in this study are worth discussion. First, one may suspect that the elevational range (0–3000 m) of Rufous-capped Babblers is not wide enough to cause altitudinal adaptation. However, this bird shows different levels of plumage UV-reflectance and brightness between lowland and montane populations, which is likely associated with different ecological environments across elevations in Taiwan [71]. In addition, another songbird—the Vinous-throated Parrotbill (*Sinosuthora webbiana*)—with a similar elevational range in Taiwan shows genomic evidence of altitudinal adaptation [72], indicating possibility of adaptive evolution of birds across the given elevational range. Second, given that we only had one highland population for the transplant experiment, some outlier expression patterns in this highland population may drive significant results when compared to the two lowland populations. Although the extent to which the observed results were driven by the possible outlier patterns is unclear, it is worth adding more highland populations in future studies. Third, as we only studied wild-caught adult birds, we cannot rule out the possibility that some observed expression differences between high- and low-altitude populations in a given common garden actually result from irreversible plastic changes that occurred during their growing stages [73]. Common garden experiments with organisms reared over generations are needed to control for the developmental plasticity. Fourth, it would be interesting to also include females to obtain a more comprehensive view regarding this bird’s altitudinal adaptation. This would be particularly relevant to the brain-based analyses because male and female birds are potentially different in some behaviors but share liver functions. However, regarding our findings including the relationships between magnitude and evolution of expression plasticity and between intra-modular connectivity and plasticity evolution, we expect similar patterns from females because these patterns are pertinent to the whole transcriptome, not only adaptive behavioral genes. Fifth, we acknowledge the need of external evidence to confirm the involvement of the ACDE genes in altitudinal adaptation (e.g., signatures of selection on genes’ regulatory regions). However, the verification of such genes would not influence the conclusions drawn from the transcriptome-wide patterns.

## Conclusions

Overall, this study (1) demonstrates the prevalence of reversing plasticity and the evolution of plasticity, (2) uncovers a positive relationship between the magnitude and evolution of gene expression plasticity, and (3) highlights the roles of genes that are peripheral to co-expression networks in Rufous-capped Babblers’ altitudinal adaptation. Being peripheral renders these genes functional or regulatory independent and thus large expression plasticity magnitude in the face of environmental changes. We speculate that when this bird’s ancestral, lowland-adapted populations colonized high mountains, the large plastic responses made the expression of these genes away from the trait optima in a new environment (i.e., reversing plasticity), potentiating subsequent genetic-based expression modifications to achieve high-altitude adaptation. Interestingly, these highly environmental-responsive genes tended to have their plasticity evolved during adaptation to high altitude. Our results also re-confirm a pattern that evolved plasticity often caused ancestral and descendant populations to have similar expression levels in the ancestral environment (Fig. 3B), suggesting that descendant populations “remember” the ancestral environment and thus may rapidly cope with it or similar environments [27]. These findings have implications in species survival in the future world with more frequent environmental changes. Specifically, more responsive genes are more likely to undergo plasticity evolution and the evolved plasticity enables the genes to show optimal expression levels in both new and old environments. Thus, more responsive genes can more easily reach optimal expression levels in a fluctuating environment once foremost plasticity evolution occurs, increasing the survival chance of species. In addition, gene expression plasticity in the liver is more responsive to environmental changes than that in the brain (Fig. 4). Following this rationale, we hypothesize that the ACDE genes in the liver play a larger role in coping with fluctuating environments than do those in the brain, and this hypothesis warrants future studies.

## Methods

### Genome resequencing and variant calling

The population structure and altitudinal colonization of the Rufous-capped Babblers in Taiwan were studied with genome-wide DNA polymorphisms generated by resequencing. We sampled individual birds from two low-altitude and two high-altitude populations (Fig. 1A), with blood collected from 10 males each population. We extracted genomic DNA using the Puregene Core Kit A (QIAGEN) following the manufacturer’s instructions. Paired-end sequencing libraries were prepared with the TruSeq DNA Sample Prep Kit v2 (Illumina) and then sequenced on an Illumina HiSeq 2500 platform with 2 × 150 base pair (bp) reads. Blood samples were collected from three additional male Rufous-capped Babblers from Hunan Province, mainland China (29.225911° N, 109.335892° E, 900 m asl) to better characterize the Taiwan-mainland relationship. We extracted genomic DNA from these additional samples using the SQ Tissue DNA Kit (OMEGA). Paired-end sequencing libraries were prepared with the MGIEasy Library Prep Kit V1.1 (MGI) and then sequenced on a BGISEQ-2000 platform with 2 × 151 bp reads. The above procedures resulted in a mean sequence coverage of 15.1 × (range = 12.2–19.6 ×) and 27.5 × (range = 27.2–27.8 ×) for the Taiwanese and mainland Chinese samples, respectively.

We removed adapter sequences in raw reads using the Trimmomatic 0.38 [74] commands ILLUMINACLIP:TruSeq3-PE.fa:2:30:10:1:true. Trimmed reads were mapped to the Babbler draft genome [75] using BWA-MEM 0.7.17 [76]. To ensure that each unique DNA fragment was only counted once, we tagged duplicated reads using the MarkDuplicates tool in the Genome Analysis Toolkit (GATK) 4.0.6 [77]. To improve variant discovery, we conducted sample-wise base quality score recalibration (BQSR) prior to the final SNP calling, as described below. First, variants were identified using GATK HaplotypeCaller [78] and BCFtools mpileup [79]. Second, concordant variants were identified by GATK, with criteria recommended by the GATK team [80] adopted to filter lower-confidence variants (QD < 2, MQ < 40, FS > 60, SOR > 3, MQRankSum <  − 12.5, or ReadPosRankSum <  − 8). Third, BQSR was carried out with GATK BaseRecalibrator based on the retained variants. The above BQSR steps were performed twice before final variant calling by BCFtools mpileup, which resulted in a total of 33,714,823 biallelic SNPs that segregated over the 43 studied Babblers.

### Relationships among populations and genetic structure estimation

We performed population genetic analyses based on putatively autosomal SNPs. For this, we mapped the Babbler’s assembled scaffolds [75] onto the (pseudo-)chromosomes of the Zebra Finch assembly (GenBank assembly accession: GCA_003957565.2) using Minimap2 [81]. Exclusion of the putative Z or W scaffolds yielded 31,964,249 putatively autosomal SNPs. We then randomly selected 10,000 SNPs to infer the inter-population phylogeny. We noted substantial divergence between Taiwan and mainland China (F_ST_ > 0.45), which rendered allelic fixation an indispensable factor in the drift processes. Accordingly, we adopted a method that takes allelic fixation into account for population tree reconstruction [55]. This method incorporates allelic fixation using a nonreversible model of genetic drift, which makes inferred evolutionary parameters “directional” and thus enables tree root identification without need to specify the outgroup.

To further assess the genetic distinctiveness among the four Taiwanese populations, we generated a second set of 10,000 randomly selected autosomal SNPs, each of which was polymorphic in the 40 Taiwanese individuals. With this data, we performed a principal component analysis (PCA) alongside an admixture model-based clustering analysis to investigate clustering of the 40 Taiwanese individuals. For the admixture analysis, we employed an ALStructure algorithm, which implements likelihood-free estimations to improve computational efficiency without compromising accuracy [82]. To confirm the robustness of the results, we repeated each of the phylogenetic analysis, PCA, and admixture analysis three times, each time with a newly generated random SNP set.

### Reciprocal transplant experiment

Another 40 male adult birds were caught in 2016 from three Taiwanese populations—10 from each of the two lowland populations, L1 and L2 (< 300 m asl), and 20 from the montane population, H (2200–2600 m asl; Fig. 1A)—and used for a reciprocal transplant experiment. This experiment did not include the H′ population due to logistical difficulties for shipping birds quickly enough to our high-altitude common garden site (h) in another mountain (Fig. 1A). Half of the captured birds (*n* = 20) from each population were randomly assigned to a common garden (l) at a low-altitude research station (Jiji, 250 m asl) and the other half (*n* = 20) to a high-altitude one (h, Hehuanshan, 3,000 m asl) belonging to the Endemic Species Research Institute. The common gardens controlled for altitude-related environmental differences such as those in temperature and oxygen pressure. Individuals were acclimated to the low- or high-altitude condition for 35–94 days (between October, 2016, and January, 2017)—except for one individual, which was acclimated for 260 days (median = 64 days; Additional file 1: Table S1 and Fig. S2)—before being sacrificed. In the common gardens, the birds were kept in 1 × 1 × 1 m cages located in rooms with many windows kept open. This setup maintains the temperature close to the outdoor temperature as well as reduces exposure of the birds to bad weather and predators. To control for potential circadian and seasonal differences in expression plasticity, all birds were sacrificed between 10 AM and 2 PM on each sampling day from January 7–20, 2017 (the coolest month of a year; Additional file 1: Fig. S2), and the entire brain, liver, and other organs were removed from each individual within 7 min. Collected tissues were immediately placed in RNAlater (Invitrogen) and incubated at 4 °C overnight, followed by storage at − 20 °C until RNA extraction.

### RNA sequencing and gene expression quantification

RNA extraction was carried out with the NucleoZOL Kit (MACHEREY–NAGEL). We identified four brain and one liver samples that showed RNA integrity number (RIN) values of < 8 in Bioanalyzer 2100 (Agilent) and excluded them from subsequent analyses. Libraries for polyA-enriched transcriptomes were constructed using TruSeq RNA Library Prep Kit v2 (Illumina); they were then sequenced on Illumina HiSeq 2500 for 2 × 125 bp paired-end reads. We sequenced 36 brain and 37 liver samples to yield 19,849,176 to 25,221,037 (mean = 22,554,647) read pairs per sample. We used the same Trimmomatic commands as for DNA resequencing to remove adapter sequences, and the command MINLEN:40 to eliminate short reads.

Two additional liver samples were sequenced in a second batch, with 2 × 201 bp reads generated on the same sequencing platform described above. From the resulting 40,139,534 and 45,645,423 read pairs, we randomly selected 22,000,000 per sample with seqtk [83] for downstream analyses to maintain similar sequencing depths across sequencing batches. When trimming these additional samples, we added the command CROP:125 in Trimmomatic to make each read ≤ 125 bp long. We showed in a sample clustering dendrogram (see the next section for details) that the two additional samples were subject to little batch effect.

For all samples, we aligned trimmed reads to the Babbler genome [75] using the splice-aware aligner HISAT2 2.1.0 [84]. When building the genome index for alignment, we incorporated splice site and exon annotations using python scripts hisat2_extract_splice_sites.py and hisat2_extract_exons.py from the HISAT2 package. We then quantified gene expression levels by counting the numbers of fragments (i.e., sequences each bookended by a pair of reads) mapped to exons of genes using featureCounts [85] with the default settings.

### Prefiltering of genes subject to confounding effects

We examined clustering of samples based on their transcriptomic profiles to identify main factors affecting gene expressions. To this end, we used the build-in method of the R package DESeq2 [86] for sample normalization. We then summarized inter-sample expression differences using Euclidean distances and performed an average-linkage analysis for sample clustering. Prior to Euclidean distance computations, we applied a variance-stabilizing data transformation [87] so that genes with contrasting expression levels would contribute approximately equally to sample clustering. The result revealed samples clustered by tissue types (Additional file 1: Fig. S3). In addition, the two liver samples sequenced in the second batch did not form their own cluster, but were well grouped with the other liver samples, indicating little batch effect.

The unequal lengths in individual birds’ acclimation time could tilt gene expressions and thus confound changes caused by factors of interest (described in the next section). We used likelihood-ratio tests implemented in DESeq2 to identify genes affected by such non-identical acclimation durations. Provided that the effect could differ when acclimating an individual to a native or a non-native environment, we identified genes under four conditions separately: lowland birds (pooled samples of L1 and L2) acclimated to low altitude, lowland birds acclimated to high altitude, montane birds (samples of H) acclimated to low altitude, and montane birds acclimated to high altitude. We binned acclimation durations into intervals of > 30, > 60, and > 90 days, and used the likelihood-ratio tests to compare models with and without including the acclimation duration as a covariate. We used the independent filtering step of DESeq2 [88] to enhance detection power and applied multiple testing corrections to control the false discovery rate at < 0.05. We then excluded genes identified in any of the four test conditions from downstream analyses for the corresponding tissues (the liver or the brain).

### Identification of adaptation-associated genes

We identified genes associated with the Rufous-capped Babbler’s high-altitude adaptation as those fulfilling two conditions: (1) genes that exhibited large and directionally concordant expression differences in the H vs. L1 and H vs. L2 population contrasts when samples were acclimated to the high-altitude garden with the montane environment, where high-altitude adaptation occurred, and (2) genes that exhibited small expression differences in the L1 vs. L2 population contrast when samples were acclimated to the low-altitude garden with the lowland environment, to which both low-altitude populations have already adapted. Given that remarkable gene expression differences between populations might represent divergence irrelevant to altitudinal adaptation or resulting from genetic drift, we incorporated the second condition to penalize genes that exhibited non-altitudinal divergence. Genes that fulfilled both conditions, referred to as the “altitudinally concordant differential expression (ACDE)” genes, were identified based on the *π*-values [57]. Given *π* =|log-fold change|× –log_10_(*P*-value), the *π*-values were always non-negative and increased as increasing magnitude and statistical significance of between-group expression differences. We derived *π*-values based on log-fold change and the *P*-value estimates from DESeq2. For each gene, we then calculated ∆*π*_1_ = *π*_(H vs. L1)_ – *π*_(L1 vs. L2)_ and ∆*π*_2_ = *π*_(H vs. L2)_ – *π*_(L1 vs. L2)_. We identified the ACDE genes as those at the top 5% for both ∆*π*_1_ and ∆*π*_2_, and meanwhile showing the same regulation directions (i.e., + /– sign) in the H vs. L1 and H vs. L2 contrasts.

### Gene function annotations and enrichment analyses

Babbler genes were functionally annotated based on their orthologous relationships with genes of Chicken, Duck, Collared Flycatcher, Turkey, and Zebra Finch. To this end, we acquired the babbler proteome predicted from the genome assembly of this species [75] alongside proteomes of the other five avian species from Ensembl v95 [89]. A longest peptide isoform per gene was kept for each species, with which multi-taxon gene orthology was inferred using the reciprocally blasting and gene clustering algorithms in SonicParanoid 1.3.0 [90]. Based on the orthology, we associated Babbler genes with gene ontology (GO) annotations of the other five species acquired from Ensembl v95 and merged as many non-replicated annotations from the latter five species as possible onto Babbler genes. Likewise, we associated Babbler genes with Kyoto Encyclopedia of Genes and Genomes (KEGG) pathway annotations of the other five birds released in June 2015.

For each tissue type studied, we examined enrichment of ACDE genes in specific GO or KEGG terms compared to the other genes expressed in the tissue, with statistical significance evaluated by one-sided Fisher exact tests. Multiple testing corrections that control false positive rates were applied under the Biological Process, Cellular Component, and Molecular Function GO main categories, separately. Within each main category, corrections were applied in a level-wise manner along the ancestor-offspring hierarchical relationships of individual terms. One false positive rate control was applied to all KEGG terms, which lacked hierarchical structure. We performed these above enrichment analyses using customized Perl scripts.

### Expression plasticity estimation for adaptation-associated genes

To infer reinforcing or reversing plasticity for each ACDE gene, we identified each gene’s directions in two expression change quantities—PC and GC (visualized in Fig. 2A). PC and GC measure the plastic response of ancestral populations and the genetic divergence between ancestral and descendant populations, respectively. We identified directions of these quantities by contrasting samples from the Rufous-capped Babbler’s extant populations (shown in Fig. 2A). Reinforcing and reversing plasticity were then inferred when obtaining consistent and opposite PC and GC directions, respectively.

Note that a gene’s expression level in the low-altitude population was used to determine both of PC and GC. Mallard et al. [58] recently demonstrated that reversing plasticity could artificially appear more prevalent than reinforcing plasticity when random sampling errors associated with the shared element falsely led to both PC ≠ 0 and GC ≠ 0. To correct for such a putative artifact, we adapted Ho and Zhang’s [24] parametric bootstrap method to our cases, which aimed to identify genes with PC ≠ 0 and GC ≠ 0 resulting from genuine differences rather than random sampling errors and was implemented as follows. For each gene, we generated two Gaussian distributions to simulate its PC and GC, respectively. The PC and GC Gaussian distributions each had a mean and a standard deviation equal to the empirical log-fold change and the associated standard error, respectively, of the PC or GC estimated by DESeq2. From the two Gaussian distributions, we drew PC and GC random samples iteratively (i.e., bootstrap replicates), with reinforcing or reversing identified by a GC-PC sample pair each time. We performed 1000 such bootstrap replicates per gene, and we concluded bootstrap-supported reinforcing or reversing once either plasticity type was obtained in ≥ 950 replicates. We conducted Gaussian distribution generations and subsequent random sampling using the rnorm function of the base R 3.6.0. We evaluated unequal frequencies between reinforcing and reversing plasticity using two-sided binomial tests against a null proportion of 0.5.

To investigate whether ACDE genes more frequently had their plasticity persisting or evolved during the bird’s high-altitude adaptation, we obtained the measure of a third expression change quantity—GC_b_—based on the population contrast shown in Fig. 3A. A binary classification between plasticity persistence and plasticity evolution was then carried out by comparing GC_b_ against GC: plasticity persistence was inferred with a GC_b_-to-GC ratio between 0.5 and < 1.5 while plasticity evolution was inferred with a ratio between − 0.5 and < 0.5 (visualized in Fig. 3B). We used two-sided binomial tests to evaluate unequal prevalence between plasticity persistence and plasticity evolution in the ACDE genes.

### Examining factors associated with evolution of expression plasticity

To examine the factors that were associated with plasticity evolution, we first used the two-sided Fisher exact test to evaluate unequal frequencies of plasticity evolution between ACDE genes exhibiting reinforcing and reversing plasticity. Secondly, to test another hypothesis that the ancestral plasticity magnitude (|PC|) determined whether the plasticity evolved, we used the Kruskal–Wallis test to assess the difference in |PC| between groups with evolved and persisting plasticity.

To dissect the above two patterns in-depth, we firstly adopted two other categorization schemes of the ACDE genes regarding the extent to which their plasticity evolved. Specifically, we used three and four equal-interval bins, respectively, to group genes according to their degree of plasticity evolution, with the gradient extending from a GC_b_-to-GC ratio ~ 1 (low degree of plasticity evolution) to a ratio ~ 0 (high degree of plasticity evolution). In the three-binned categorization, we grouped genes by GC_b_-to-GC ratios between 0.75 and < 1.25, between 0.25 and < 0.75, and between − 0.25 and < 0.25. In the four-binned categorization, we grouped genes by GC_b_-to-GC ratios between 0.83 and < 1.17, between 0.50 and < 0.83, between 0.17 and < 0.50, and between − 0.17 and < 0.17 (both categorizations are visualized in Additional file 1: Fig. S4A). Secondly, we tested the two focal patterns with genes’ plasticity evolution measured on a continuous scale. To this end, we used DESeq2 to quantify the absolute difference between the lowland and montane populations in their reaction norms, estimated by the population × acclimation environment interaction in the linear model (visualized in Additional file 1: Fig. S5A).

We also examined whether the hypothesis that |PC| determined the evolution of plasticity held in the rest of the Rufous-capped Babbler’s genes as in the ACDE genes. We pooled samples from the two low-altitude populations for acquiring |PC| and the above plasticity evolution measures for each gene. The sample pooling was to be consistent with the expression module analyses described in the following section.

We used the 2 × C Fisher exact test to evaluate inequality among plasticity evolution categories in their proportions exhibiting reinforcing and reversing plasticity (C = 3 and 4 in cases of three and four evolution categories, respectively). This was followed with two-sided 2 × 2 Fisher exact tests for post hoc comparisons between pairwise categories. Similarly, we used the Kruskal–Wallis test to evaluate inequality among evolution categories in |PC|, followed with post hoc pairwise comparisons using two-sided Dunn tests. We implemented Fisher exact tests in the R package rstatix [91], Kruskal–Wallis tests in the base R, and Dunn tests in the R package FSA [92]. Multiple testing corrections were applied to post hoc Fisher and Dunn tests. In cases where plasticity evolution was scaled continuously, we used the Kruskal–Wallis test to evaluate the difference between reinforcing and reversing plasticity genes in their plasticity evolution; we used the one-sided Spearman correlation test in the base R to examine the relationship between |PC| and plasticity evolution.

### Transcriptome-wide co-expression analyses

We demonstrated the association between |PC| and plasticity evolution to be transcriptome-wide rather than limited to the ACDE genes (see “Results”). We hypothesized both |PC| and plasticity evolution to be dependent on the level of gene–gene interactions in the ancestral population, leading to the observed positive relationship between |PC| and plasticity evolution. To test this hypothesis, we first applied the WGCNA analyses [61, 62] to genes of the whole transcriptomes to delimit groups of expressionally interacting genes (co-expression modules) in the Rufous-capped Babbler’s lowland populations L1 and L2. We pooled samples from the two low-altitude populations to fulfill the minimum sample size for this analysis [93]. Briefly, the WGCNA grouped genes based on their correlations in expressions. By raising the absolute values of the correlations to a power *β* > 1 (referred to as “gene adjacency”), the analysis gives weight to gene clustering with high correlations. Prior to the analysis, we filtered out genes with many zero RNA fragment counts because such genes would show spuriously high correlations with one another; specifically, we removed genes showing a median absolute deviation value = 0. We quantified correlations between genes using the biweight midcorrelation, which is robust to the presence of outlier samples [94]; we set 0.1 as the maximum percentile of data that can be considered outliers. We performed “signed” WGCNA analyses such that genes were clustered based on positive correlations. We chose power *β* values under the following considerations: (1) the fit to the scale-free topology model > 0.8; (2) a large *β* value to avoid clustering based on spurious correlations between genes; (3) clustering supported by a reasonable mean gene connectivity value (~ 100), which necessarily decreases with increasing *β*. Consequently, we selected *β* = 12 and 18 for the brain- and liver-based WGCNA analyses (Additional file 1: Fig. S13A). We performed average-linkage clustering of the genes based on their topological overlaps derived from the adjacency measures. We then applied the dynamic tree cut algorithm for module delimitations with the minimum module size and the deep-split parameters set to 30 and 2, respectively.

To test the dependence of plasticity evolution on gene–gene interactions, we conducted three examinations. Firstly, we used the one-sided Spearman correlation test to evaluate correlations between the module size and the proportion of genes with plasticity evolution in the module. However, genes may differ in the levels of gene–gene interactions even when they occur in modules with similar sizes. Considering this possible scenario, we obtained measures of the intra-modular connectivity (*k*_IM_) of individual genes, calculated for each gene as the sum of its adjacencies. For the second examination, we subdivided genes’ plasticity evolution into two, three or four levels and then used Kruskal–Wallis tests to evaluate inequalities among evolution categories in the *k*_IM_ values. In cases with three or four evolution categories, Dunn tests with multiple testing corrections were used for post hoc pairwise comparisons. For the final examination, we measured genes’ plasticity evolution on a continuous scale and used the one-sided Spearman correlation test to evaluate the hypothesized negative correlation between *k*_IM_ and the plasticity evolution. In the second and third examinations, we adopted both raw and scaled *k*_IM_. We scaled *k*_IM_ by its maximum value per module to render values all between zero and one; we aimed to confirm the resultant patterns insensitive to different *k*_IM_ scales across modules of various sizes.

### ACDE vs. non-ACDE genes

We used Kruskal–Wallis tests to evaluate differences between ACDE and non-ACDE genes in (1) *k*_IM_, (2) ancestral plasticity magnitude (= plasticity magnitude in the lowland populations), and (3) the extent to which plasticity evolved. For (2) and (3), we derived focal quantities with the two lowland populations L1 and L2 pooled. We used the continuous measure of genes’ plasticity evolution for (3).

### Statistical significance

Results from all statistical tests were regarded significant whenever obtaining nominal *P*-values (or multiple testing adjusted *P*-values) < 0.05. We applied Benjamini and Hochberg’s [95] procedure for multiple testing corrections throughout this study.

## Supplementary Information

**Additional file 1: Fig. S1.** ALStructure inferred genetic clustering of the 40 Rufous-capped Babblers from the four Taiwanese populations. **Fig. S2.** Ambient temperature in the two common gardens and acclimation durations of the 40 Rufous-capped Babblers. **Fig. S3.** Dissimilarity in the transcriptome-wide gene expression profile among samples. **Fig. S4.** Associations between ACDE genes’ ancestral plasticity directions and their plasticity evolution levels. **Fig. S5.** Associations between ACDE genes’ ancestral plasticity directions and their plasticity evolution degree (continuously scaled). **Fig. S6.** Plasticity evolution tends to occur in ACDE genes with bootstrap support. **Fig. S7.** Complement of Fig. 4 with ACDE genes exhibiting reinforcing ancestral plasticity. **Fig. S8.** Associations between ACDE genes’ ancestral plasticity magnitude (|PC|) and their plasticity evolution levels. **Fig. S9.** Associations between |PC| and the continuous-scaled plasticity evolution degree in the ACDE genes. **Fig. S10.** Associations between |PC| and the continuous-scaled plasticity evolution degree in the bootstrap-supported ACDE genes. **Fig. S11.** Associations between |PC| and plasticity evolution levels in the non-ACDE Rufous-capped Babbler genes. **Fig. S12.** Associations between |PC| and the continuous-scaled plasticity evolution degree in the non-ACDE Rufous-capped Babbler genes. **Fig. S13.** WGCNA for delimitating groups of expressionally interacting genes. **Fig. S14.** Differences in the unscaled intra-modular connectivity among gene categories that show different plasticity evolution levels. **Fig. S15.** Differences in the scaled intra-modular connectivity among gene categories that show different plasticity evolution levels. **Fig. S16.** Rufous-capped Babbler genes’ expression plasticity evolution is negatively associated with the level of intra-modular connectivity. **Fig. S17.** ACDE genes have lower levels of intra-modular connectivity than non-ACDE genes. **Fig. S18.** ACDE genes exhibit larger magnitude of ancestral plasticity and higher degree of plasticity evolution than non-ACDE genes. **Table S1.** Acclimation durations and RNA fragment counts of the 40 studied Rufous-capped Babblers. **Table S2.** Enriched Biological Process gene ontologies and Kyoto Encyclopedia of Genes and Genomes terms for the liver-expressed ACDE genes.

## Data Availability

Genomic SNP data, Perl scripts for GO and KEGG enrichment analyses, and R codes used are available in the figshare repository (https://doi.org/10.6084/m9.figshare.17001145.v2) [96]. RNA-seq reads generated by this study are deposited in the NCBI database under the accession number PRJNA941087 [97].

## Abbreviations

ACDE: Altitudinally concordant differential expression
asl: Above sea level
bp: Base pair
BQSR: Base quality score recalibration
F_ST_: Fixation index
GATK: Genome Analysis Toolkit
GC: Genetic-based expression change
GO: Gene Ontology
KEGG: Kyoto Encyclopedia of Genes and Genomes
*k*_IM_: Intra-modular connectivity
Ma: Million years ago
NASA: National Aeronautics and Space Administration
PC: Plastic expression change
PCA: Principal component analysis
RI: Reinforcing plasticity
RIN: RNA integrity number
RV: Reversing plasticity
SNP: Single-nucleotide polymorphism
TC: Total expression change
WGCNA: Weighted gene co-expression network analysis
