# Supplementary material for: Weak gene–gene interaction facilitates the evolution of gene expression plasticity
Source: BMC Biol. 2023 Mar 20;21:57. doi: 10.1186/s12915-023-01558-6 (PMC10029303; doi:10.1186/s12915-023-01558-6)
Supplement: Supplementary file 1 — Additional file 1: Fig. S1. ALStructure inferred genetic clustering of the 40 Rufous-capped Babblers from the four Taiwanese populations. Fig. S2. Ambient temperature in the two common gardens and acclimation durations of the 40 Rufous-capped Babblers. Fig. S3. Dissimilarity in the transcriptome-wide gene expression profile among samples. Fig. S4. Associations between ACDE genes’ ancestral plasticity directions and their plasticity evolution levels. Fig. S5. Associations between ACDE genes’ ancestral plasticity directions and their plasticity evolution degree (continuously scaled). Fig. S6. Plasticity evolution tends to occur in ACDE genes with bootstrap support. Fig. S7. Complement of Fig. 4 with ACDE genes exhibiting reinforcing ancestral plasticity. Fig. S8. Associations between ACDE genes’ ancestral plasticity magnitude (|PC|) and their plasticity evolution levels. Fig. S9. Associations between |PC| and the continuous-scaled plasticity evolution degree in the ACDE genes. Fig. S10. Associations between |PC| and the continuous-scaled plasticity evolution degree in the bootstrap-supported ACDE genes. Fig. S11. Associations between |PC| and plasticity evolution levels in the non-ACDE Rufous-capped Babbler genes. Fig. S12. Associations between |PC| and the continuous-scaled plasticity evolution degree in the non-ACDE Rufous-capped Babbler genes. Fig. S13. WGCNA for delimitating groups of expressionally interacting genes. Fig. S14. Differences in the unscaled intra-modular connectivity among gene categories that show different plasticity evolution levels. Fig. S15. Differences in the scaled intra-modular connectivity among gene categories that show different plasticity evolution levels. Fig. S16. Rufous-capped Babbler genes’ expression plasticity evolution is negatively associated with the level of intra-modular connectivity. Fig. S17. ACDE genes have lower levels of intra-modular connectivity than non-ACDE genes. Fig. S18. ACDE genes exhibit larger magnitude of ances [file 12915_2023_1558_MOESM1_ESM.docx]

**Weak gene-gene interaction facilitates the evolution of gene expression plasticity**

Hao-Chih Kuo, Cheng-Te Yao, Ben-Yang Liao, Meng-Pin Weng, Feng Dong, Yu-Cheng Hsu, Chih-Ming Hung

**Supplementary Materials:**

Supplementary Figures 1-18

Supplementary Tables 1-2


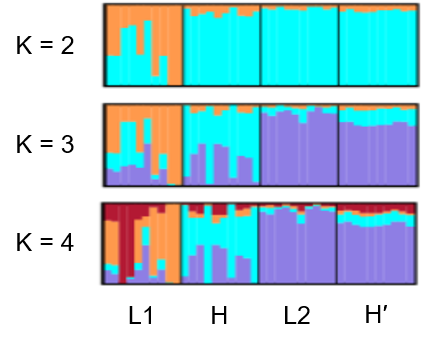


**Figure S1. ALStructure inferred genetic clustering of the 40 Rufous-capped Babblers from the four Taiwanese populations.** Inferences are carried out with numbers of genetic clusters (K) predetermined as two, three and four. Individual birds are presented as bars that are proportionally colored to show their inferred ancestries from the K clusters. At K = 3, the recovered clusters broadly correspond to populations L1, H and the combination of L2 and H′, respectively. The adding cluster is found in L1 when increasing K from three to four.


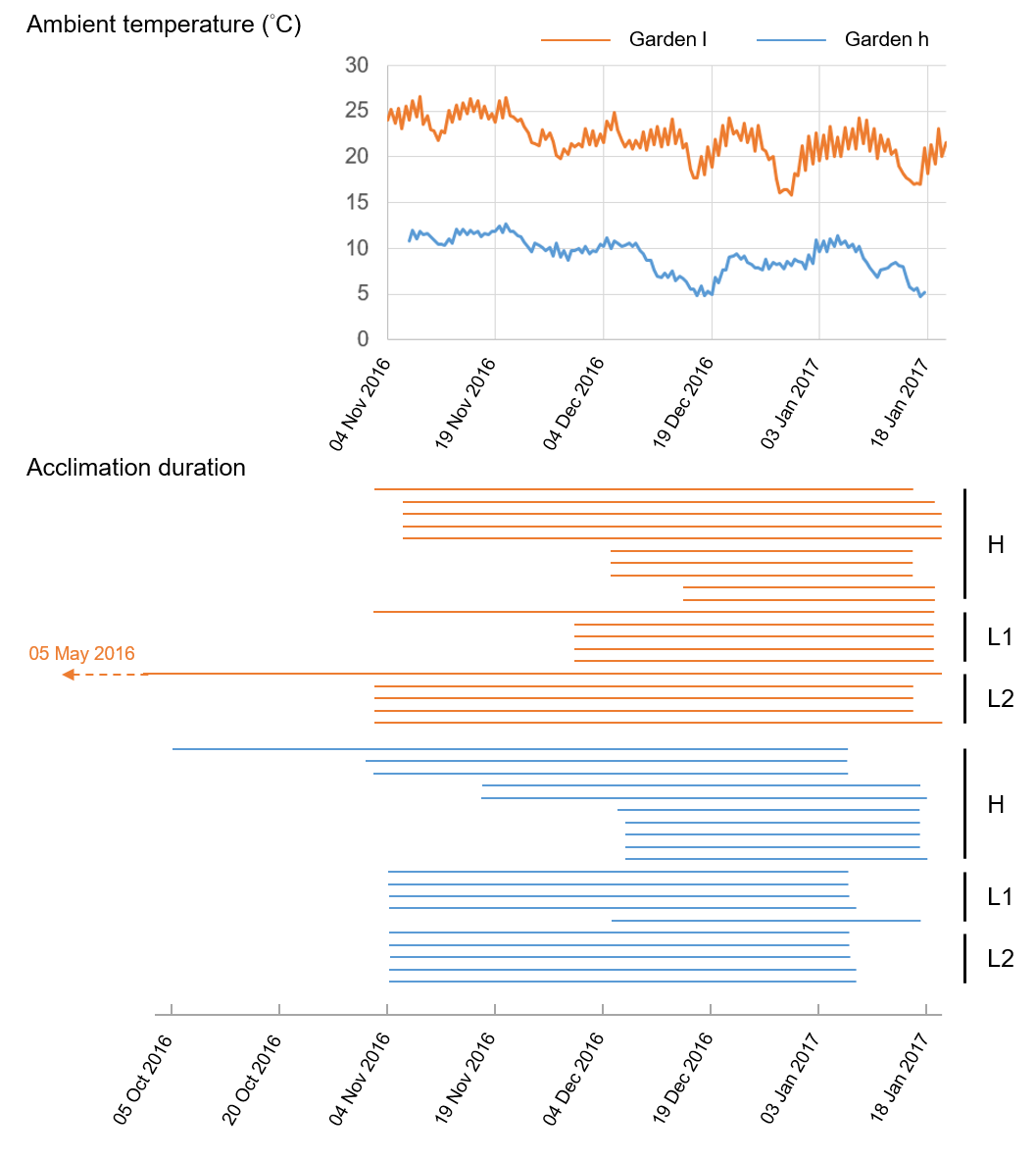


**Figure S2. Ambient temperature in the two common gardens and acclimation durations of the 40 Rufous-capped Babblers.** L1 and L2 denote the two low-altitude populations while H denote the high-altitude population.


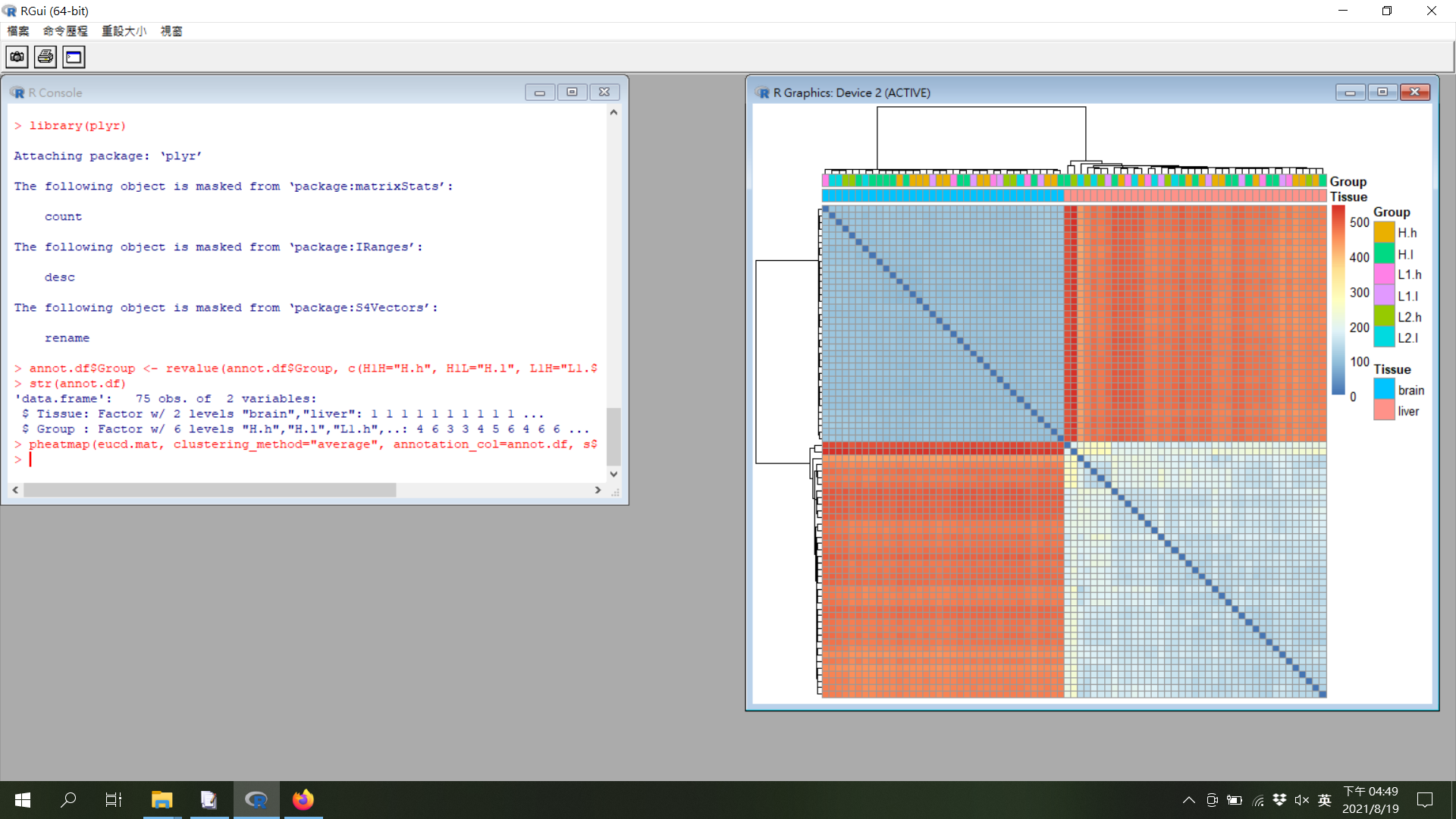


**Figure S3. Dissimilarity in the transcriptome-wide gene expression profile among samples.** Dissimilarity—quantified by Euclidean distances—occurs mainly between tissue types and to much less extent between source populations (L1, L2 and H) or acclimation environments (h and l for high- and low-altitude environments, respectively). A variance stabilizing transformation is applied prior to dissimilarity calculation to minimize the dependence of inter-sample variances to gene expression levels. The clustering dendrograms are built with an average-linkage algorithm.


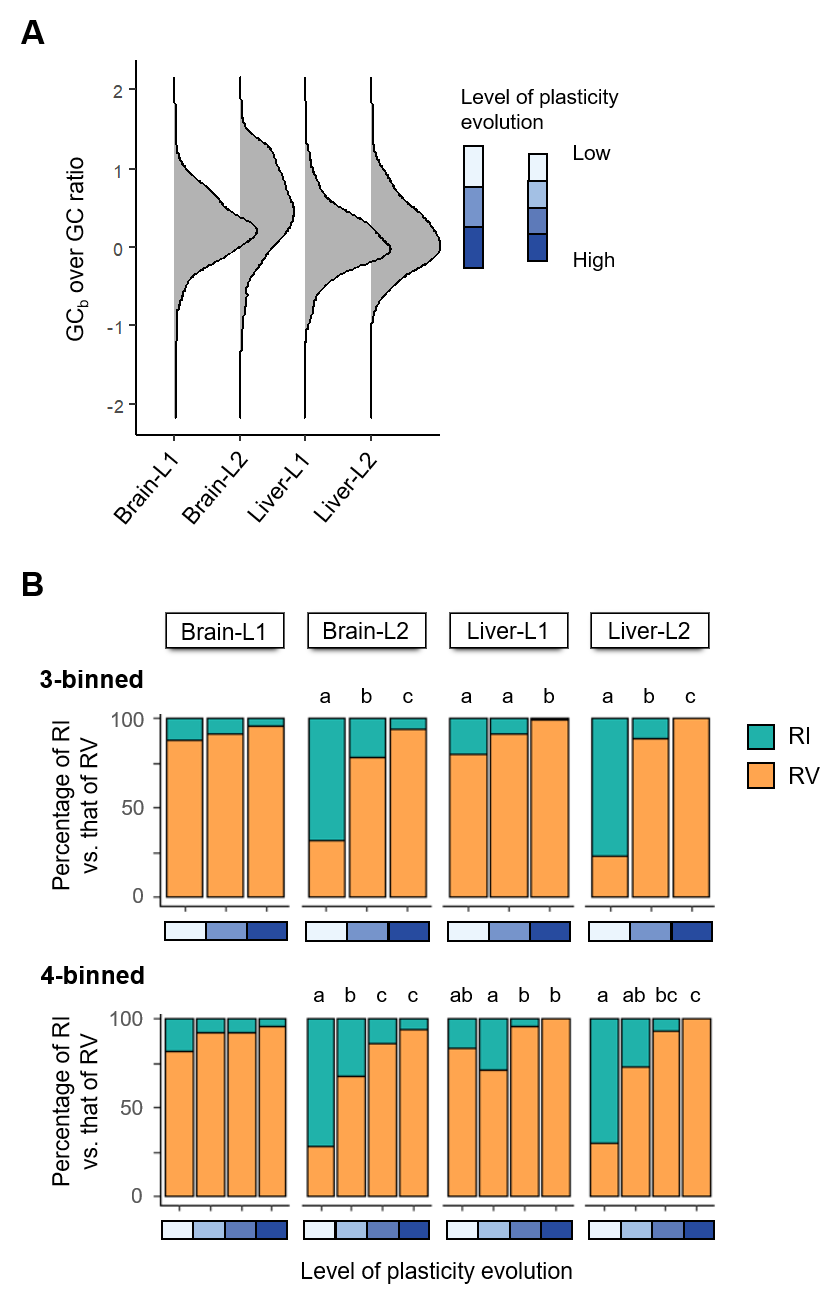


**Figure S4. Associations between ACDE genes’ ancestral plasticity directions and their plasticity evolution levels.** (**A**) Three or four bins are used to categorize genes according to the extent to which their expression plasticity evolves (see Fig. 3A for definitions of GC and GC_b_; see Methods for binning details). (**B**) In general, greater levels of plasticity evolution are associated with higher proportions of genes exhibiting ancestral plasticity direction as reversing (RV) than as reinforcing (RI). The dependence between the two factors is supported by 2 × 3 and 2 × 4 Fisher exact tests (P < 0.05) except in the brain-L1 condition. Lowercase letters above the bars denote the post-hoc pairwise comparison results by 2 × 2 Fisher exact tests, with different letters indicating significant proportional differences after the multiple testing corrections (adjusted P < 0.05).


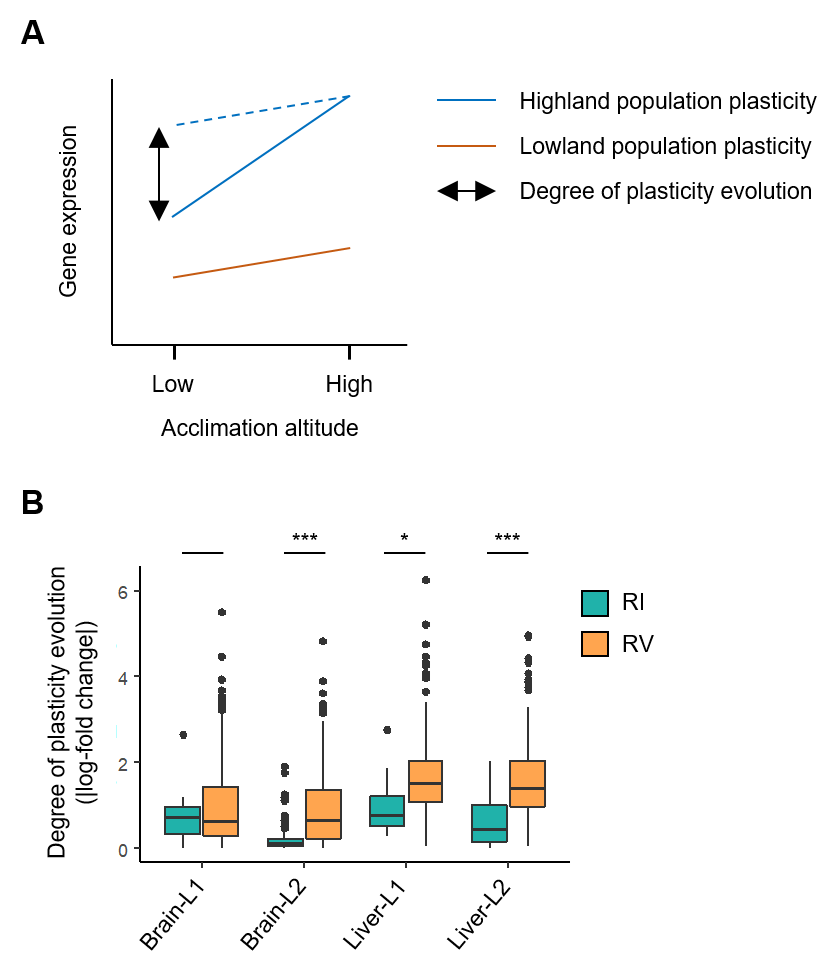


**Figure S5. Associations between ACDE genes’ ancestral plasticity directions and their plasticity evolution degree (continuously scaled).** (**A**) The absolute magnitude of divergence (|log-fold change|) between the highland and the lowland populations in their expression plasticity is used as a continuous measure of the plasticity evolution degree. The blue dash line presents the plasticity in the highland population were it not divergent from the lowland population plasticity. (**B**) In all except the brain-L1 condition, genes exhibiting reversing (RV) ancestral plasticity undergo significantly higher degree of plasticity evolution than do those exhibiting reinforcing (RI) ancestral plasticity (Kruskal-Wallis tests; ***, * and the blank indicate P < 0.001, < 0.05 and > 0.05, respectively).


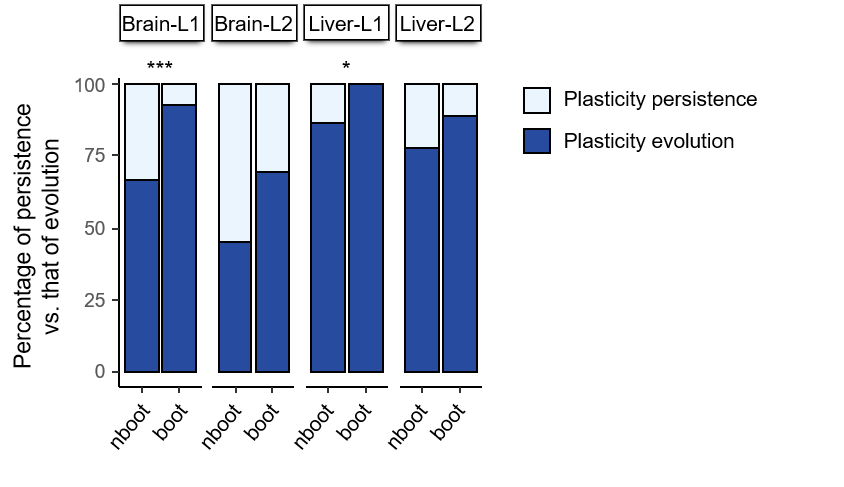


**Figure S6. Plasticity evolution tends to occur in ACDE genes with bootstrap support.** The bootstrap-supported genes (boot) consistently show higher proportions of plasticity evolution than do those without bootstrap support (nboot) in all four conditions. The proportional differences gain statistical significance in two of the four conditions (two-sided Fisher exact tests; ***, * and the blank indicate P < 0.001, < 0.05 and > 0.05, respectively).


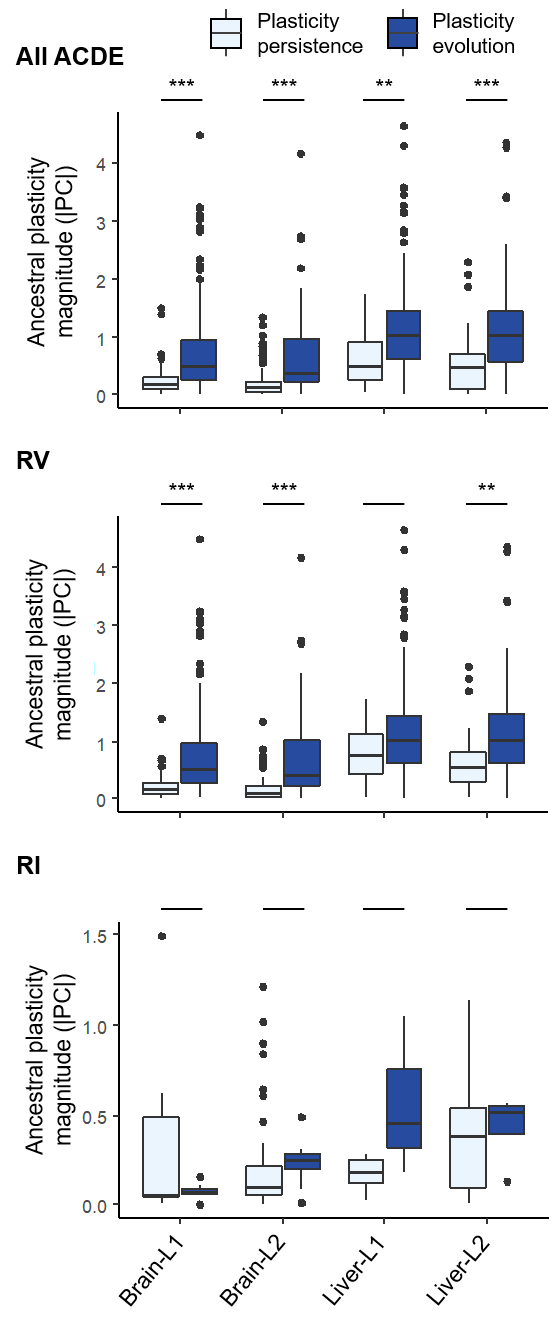


**Figure S7. Complement of Figure 4 with ACDE genes exhibiting reinforcing (RI) ancestral plasticity.**


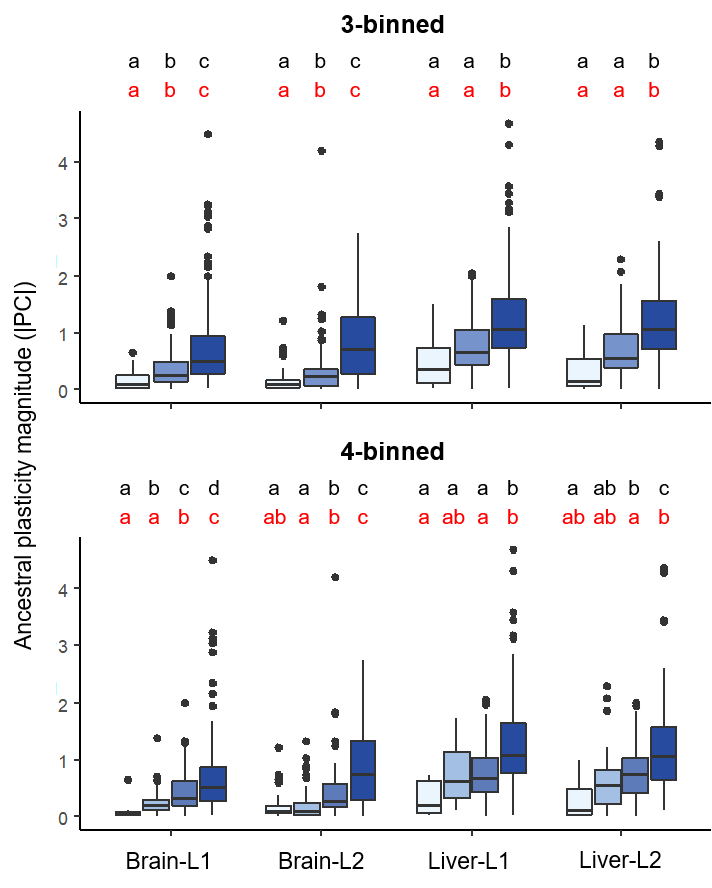


**Figure S8. Associations between ACDE genes’ ancestral plasticity magnitude (|PC|) and their plasticity evolution levels.** Three or four bins are used to categorize genes according to the extent to which their expression plasticity evolves (darker blues for higher evolution levels). Comparisons are made for all ACDE genes and the subset exhibiting reversing plasticity; comparisons based on ACDE genes that exhibit reinforcing plasticity are not made due to lacks of such genes in one or more plasticity-evolution categories. Significant inter-category |PC| differences are detected by Kruskal-Wallis tests in all cases (P < 0.001 for all ACDE genes, P < 0.01 for the subset exhibiting reversing plasticity). Lowercase letters above the boxplots denote the post-hoc pairwise comparison results by two-sided Dunn tests, with different letters indicating significant |PC| differences after the multiple testing corrections (adjusted P < 0.05; black and red letters denote results obtained with all ACDE genes and with the subset exhibiting reversing plasticity, respectively).


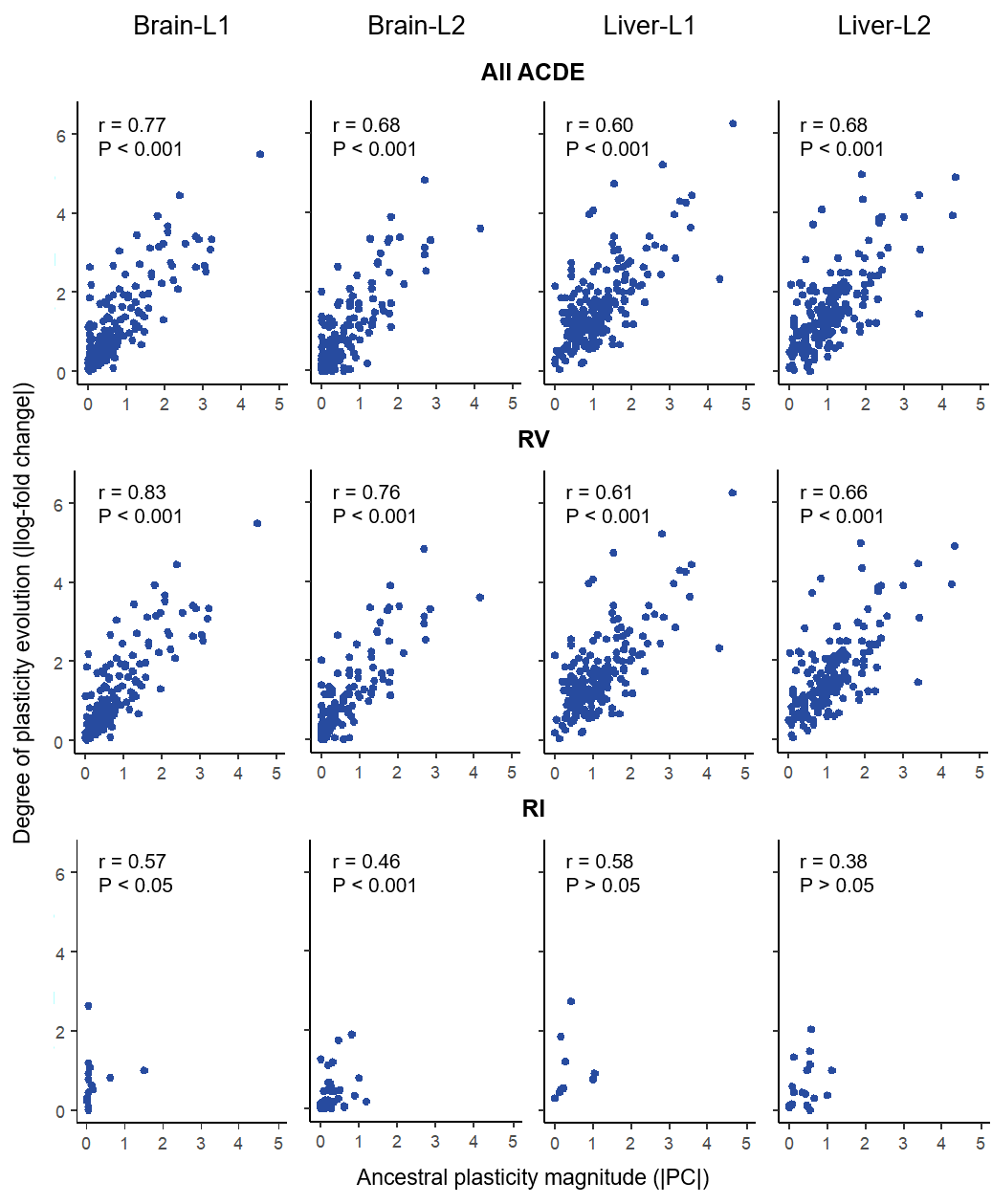


**Figure S9. Associations between |PC| and the continuous-scaled plasticity evolution degree in the ACDE genes.** The degree of plasticity evolution is quantified as in Fig. S5A. One-sided Spearman correlation tests are used to evaluated the hypothesized positive association between |PC| and plasticity evolution degree. Results are shown for all ACDE genes as well as for the subsets that exhibit either reversing (RV) or reinforcing (RI) plasticity.

**
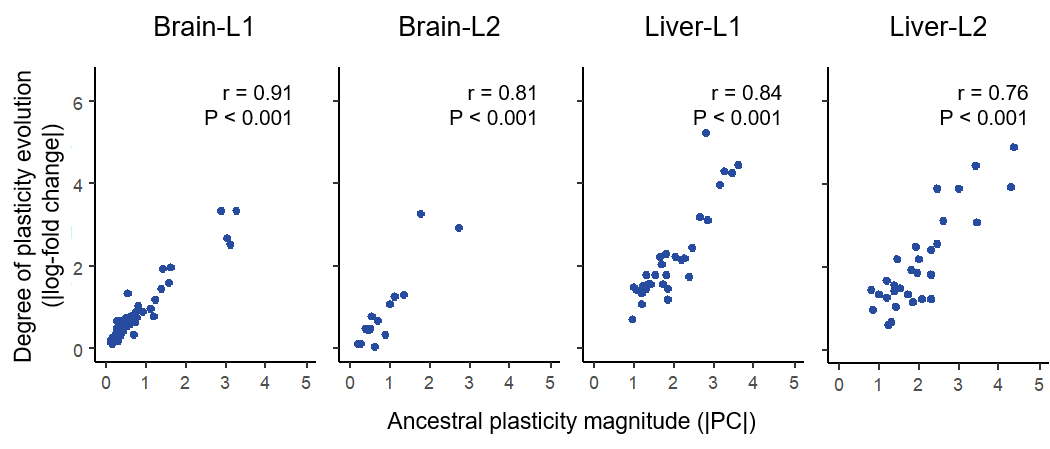
**

**Figure S10. Associations between |PC| and the continuous-scaled plasticity evolution degree in the bootstrap-supported ACDE genes.** The degree of plasticity evolution is quantified as in Fig. S5A. One-sided Spearman correlation tests are used to evaluated the hypothesized positive association between |PC| and the plasticity evolution degree.


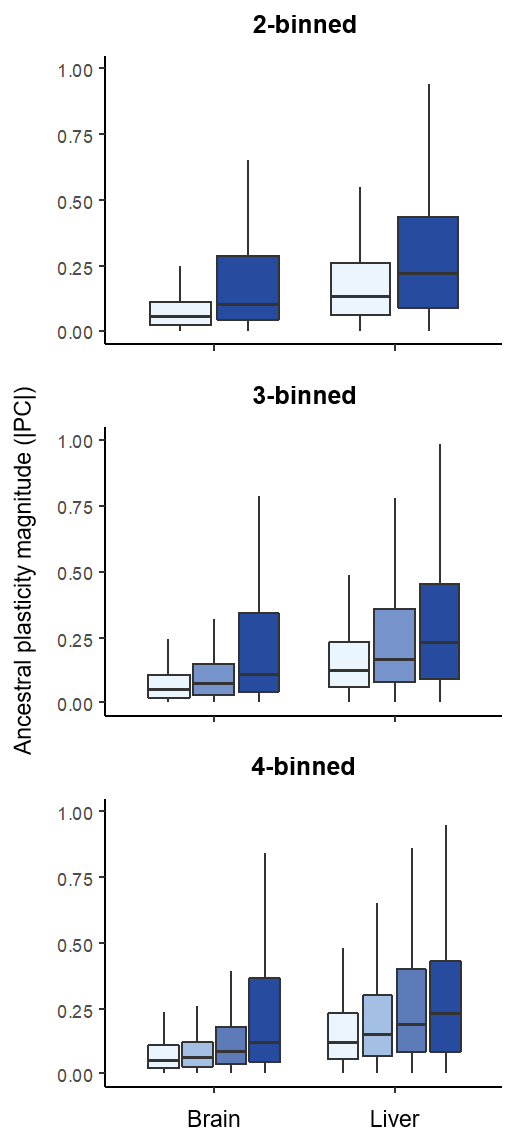


**Figure S11. Associations between |PC| and plasticity evolution levels in the non-ACDE Rufous-capped Babbler genes.** Two, three or four bins are used to categorize genes according to the extent to which their expression plasticity evolves (darker blues for higher evolution levels). Kruskal-Wallis tests detect significant inter-category |PC| differences in all cases (P < 0.001). After the multiple testing corrections, two-sided Dunn tests further detect significant |PC| differences between all pairs of plasticity-evolution categories in all cases (adjusted P < 0.05). The plots are shown with outlier |PC| points removed for clarity.

**
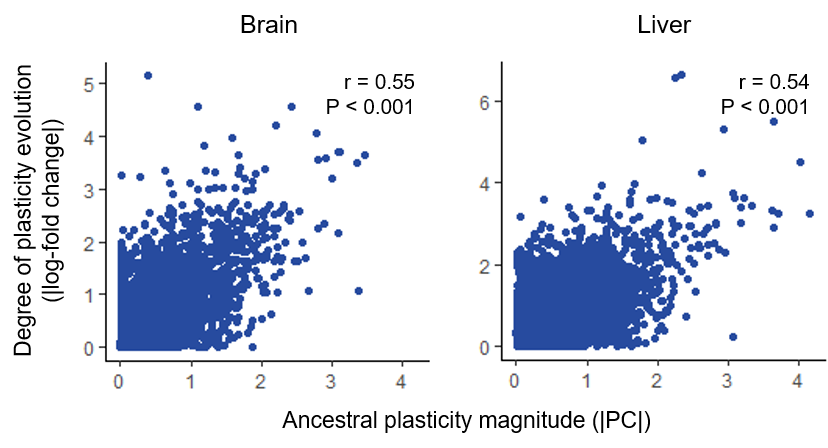
**

**Figure S12. Associations between |PC| and the continuous-scaled plasticity evolution degree in the non-ACDE Rufous-capped Babbler genes.** The degree of plasticity evolution is quantified as in Fig. S5A. One-sided Spearman correlation tests are used to evaluated the hypothesized positive association between |PC| and the plasticity evolution degree.


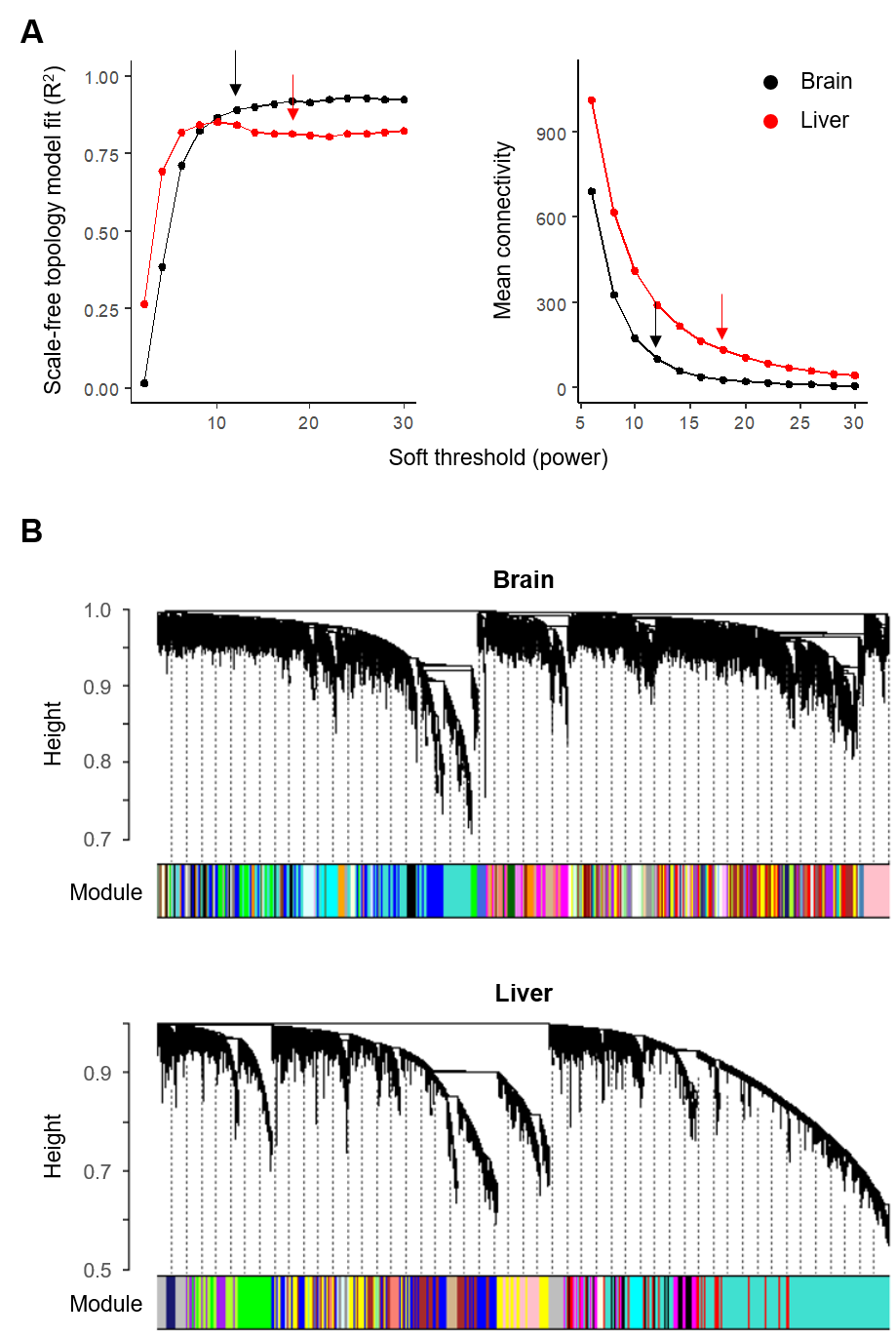


**Figure S13. Weighted gene co-expression network analyses (WGCNA) for delimitating groups of expressionally interacting genes (co-expression modules).** (**A**) Scale-free topology model fits and mean connectivity of a gene to the others under different soft threshold parameter values (β). We selected β = 12 and 18 for the brain- and liver-based network building, respectively (indicated by arrows); we selected a smaller β for the brain-based analysis because in this case the mean connectivity dropped more quickly with increasing β (see Materials and Methods for our β selection criteria). (**B**) Average-linkage clustering of the genes together with co-expression modules (differently colored based on their identities) delimited along the dendrograms using the dynamic tree cut algorithm. After excluding individual genes that are unable to be assigned into modules (colored in grey) for their being distantly related to all the other genes, the brain- and liver-based analyses delimited 67 and 20 modules, respectively.


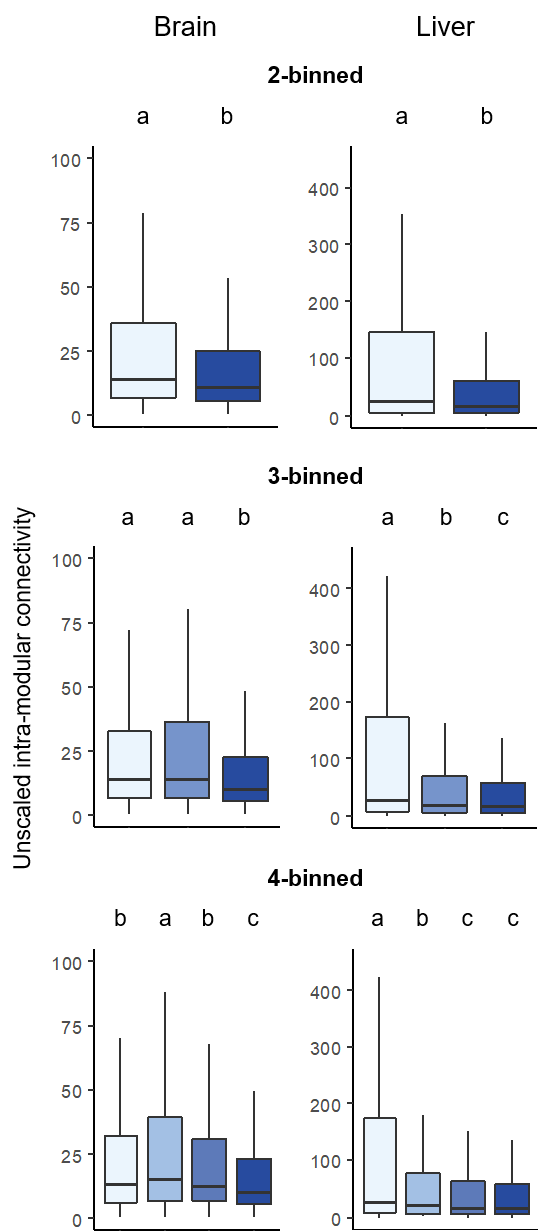


**Figure S14. Differences in the unscaled intra-modular connectivity among gene categories that show different plasticity evolution levels.** Two, three or four bins are used to categorize Rufous-capped Babbler genes according to the extent to which their expression plasticity evolves (darker blues for higher evolution levels). Comparisons are made with the unscaled intra-modular connectivity (*k*_IM.US_). Significant inter-category *k*_IM.US_ differences are detected by Kruskal-Wallis tests in all cases (P < 0.001). Lowercase letters above the boxplots denote the post-hoc pairwise comparison results by two-sided Dunn tests, with different letters indicating significant *k*_IM.US_ differences after the multiple testing corrections (adjusted P < 0.05). The plots are shown with outlier connectivity values removed for clarity.


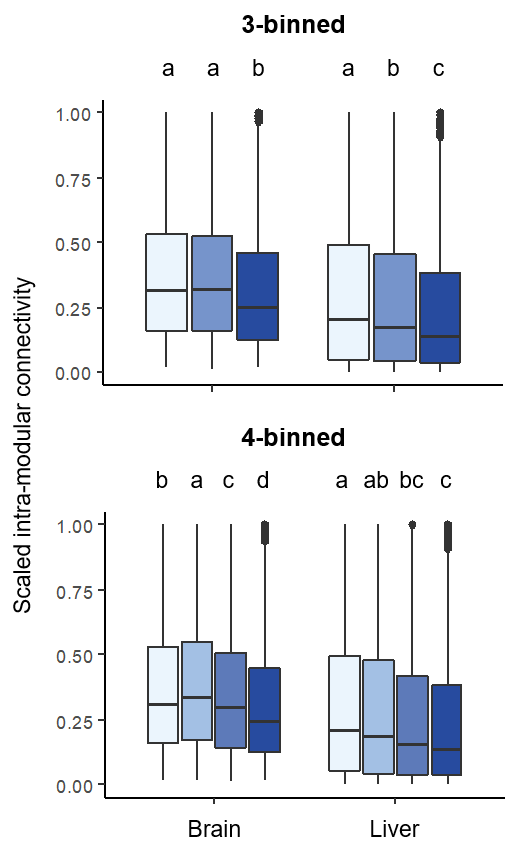


**Figure S15. Differences in the scaled intra-modular connectivity among gene categories that show different plasticity evolution levels.** Three or four bins are used to categorize Rufous-capped Babbler genes according to the extent to which their expression plasticity evolves (darker blues for higher evolution levels). Intra-modular connectivity values are scaled by the maxima of the corresponding modules (*k*_IM.S_). Significant inter-category *k*_IM.S_ differences are detected by Kruskal-Wallis tests in all cases (P < 0.001). Lowercase letters above the boxplots denote the post-hoc pairwise comparison results by two-sided Dunn tests, with different letters indicating significant *k*_IM.S_ differences after the multiple testing corrections (adjusted P < 0.05).


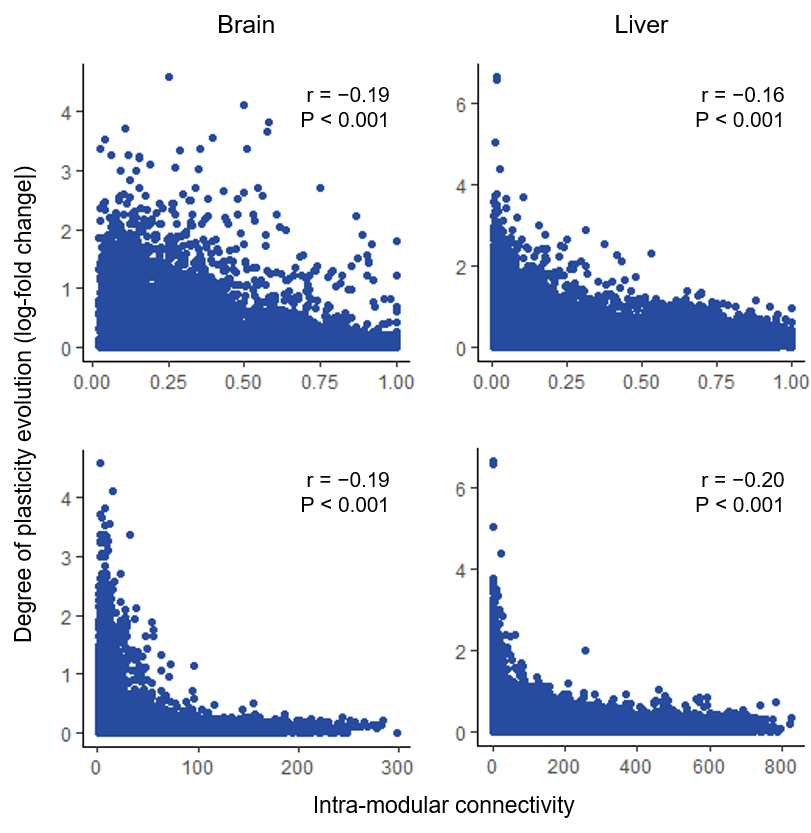


**Figure S16. Rufous-capped Babbler genes’ expression plasticity evolution is negatively associated with the level of intra-modular connectivity.** Genes have degree of plasticity evolution quantified as in Fig. S5A. One-sided Spearman correlation tests support the negative associations (P < 0.001) regardless of whether the connectivity values are scaled by the maxima of the corresponding modules (upper row) or not (lower row).

**
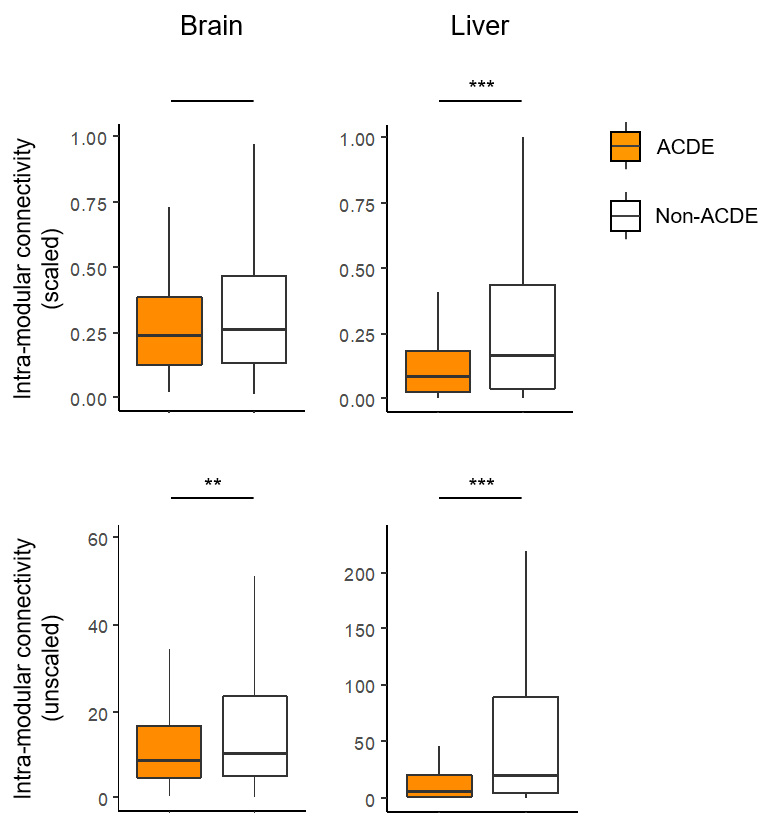
**

**Figure S17. ACDE genes have lower levels of intra-modular connectivity than non-ACDE genes.** Kruskal-Wallis tests are used to evaluate inter-group differences (***, ** and the blank indicate P < 0.001, < 0.01 and > 0.05, respectively). The plots are shown with outlier connectivity values removed for clarity.

**
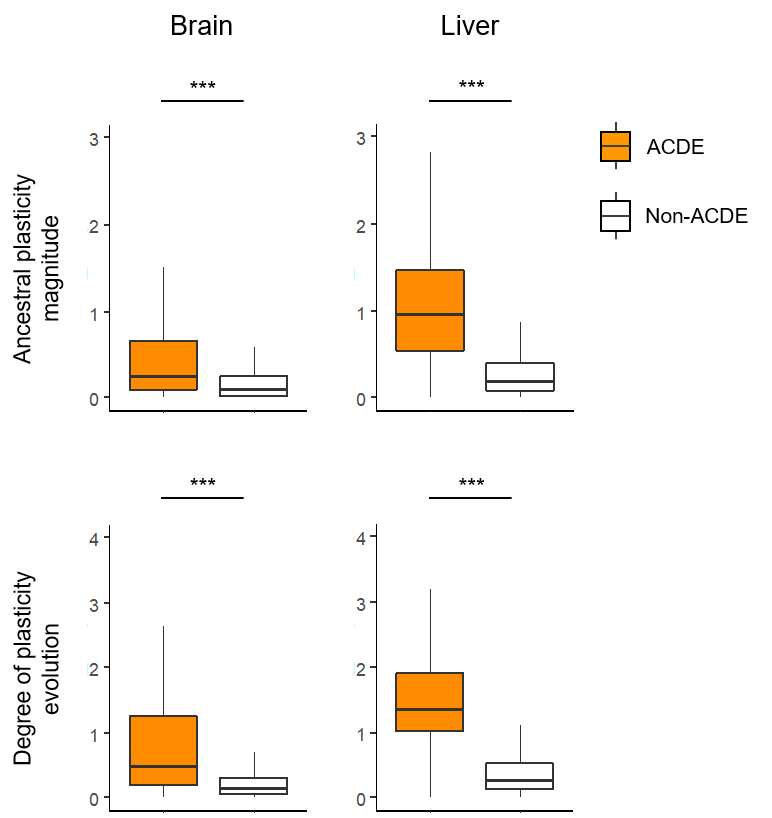
**

**Figure S18. ACDE genes exhibit larger magnitude of ancestral plasticity and higher degree of plasticity evolution than non-ACDE genes.** Plasticity evolution is quantified as in Fig. S5A. Kruskal-Wallis tests are used to evaluate inter-group differences (*** indicates P < 0.001). The plots are shown with outlier points removed for clarity.

| **Table S1. Acclimation durations (DUR) and RNA fragment counts (FCNT) of the 40 studied Rufous-capped Babblers.** | | | | | |
| --- | --- | --- | --- | --- | --- |
|  |  |  | DUR  (day) | FCNT | |
| Individual | Population | Garden |  | Brain | Liver |
| SRU201 | H | h | 94 | 8,490,791 | 11,592,963 |
| SRU213 | H | h | 66 | 8,634,181 | 12,686,060 |
| SRU246 | H | h | 61 | 8,563,156 | 13,321,118 |
| SRU248 | H | h | 62 | 8,755,168 | 12,680,270 |
| SRU272 | H | h | 42 | 8,864,974 | 12,557,987 |
| SRU273 | H | h | 41 | 8,076,358 | 12,828,370 |
| SRU274 | H | h | 41 | 8,581,548 | 12,658,140 |
| SRU276 | H | h | 42 | 8,980,926 | 13,855,978 |
| SRU277 | H | h | 41 | 9,350,833 | 12,901,871 |
| SRU301 | H | h | 67 | 8,577,363 | 11,335,223 |
| SRU203 | H | l | 74 | 9,394,171 | 12,625,989 |
| SRU208 | H | l | 75 | 8,699,378 | 13,110,620 |
| SRU238 | H | l | 35 | 8,363,275 | 11,469,649 |
| SRU240 | H | l | 75 | 8,433,060 | 13,709,572 |
| SRU242 | H | l | 75 | 8,856,830 | 11,047,405 |
| SRU243 | H | l | 35 | 7,907,592 | 12,278,598 |
| SRU250 | H | l | 75 | 8,982,754 | 10,899,406 |
| SRU267 | H | l | 42 | 7,779,836 | 12,348,074 |
| SRU268 | H | l | 42 | 8,215,655 | 13,685,418 |
| SRU271 | H | l | 42 | 9,772,708 | 12,185,519 |
| SRU229 | L1 | h | 64 | n/a | 12,768,869 |
| SRU231 | L1 | h | 64 | 7,394,905 | n/a |
| SRU232 | L1 | h | 65 | 8,127,511 | 12,294,107 |
| SRU233 | L1 | h | 64 | 8,341,653 | 12,387,411 |
| SRU261 | L1 | h | 43 | 7,974,846 | 12,041,976 |
| SRU234 | L1 | l | 78 | n/a | 12,550,656 |
| SRU259 | L1 | l | 50 | 8,500,241 | 14,611,881 |
| SRU263 | L1 | l | 50 | 8,215,124 | 10,882,946 |
| SRU264 | L1 | l | 50 | 8,712,433 | 11,759,042 |
| SRU265 | L1 | l | 50 | 8,051,083 | 12,081,625 |
| SRU084 | L2 | h | 64 | n/a | 10,904,017 |
| SRU089 | L2 | h | 65 | 8,242,483 | 11,065,455 |
| SRU101 | L2 | h | 64 | 8,335,974 | 14,085,968 |
| SRU107 | L2 | h | 65 | 9,090,424 | 12,923,173 |
| SRU108 | L2 | h | 64 | 9,015,997 | 12,891,658 |
| SRU087 | L2 | l | 79 | n/a | 11,916,667 |
| SRU099 | L2 | l | 260 | 8,865,259 | 12,561,011 |
| SRU222 | L2 | l | 75 | 8,396,141 | 12,597,444 |
| SRU224 | L2 | l | 75 | 8,261,181 | 12,262,265 |
| SRU225 | L2 | l | 75 | 8,340,555 | 14,880,381 |

| **Table S2. Enriched Biological Process (BP) gene ontologies and Kyoto Encyclopedia of Genes and Genomes (KEGG) terms for the liver-expressed ACDE genes.** Significance of enrichment is evaluated by one-sided Fisher exact tests followed by multiple testing corrections. The (enrichment) ratio is the number of ACDE genes belonging to a specific term (n1) divided by the term’s all genes. | | | | | |
| --- | --- | --- | --- | --- | --- |
|  | Term ID | Description | n1 | ratio | Adjusted P |
| BP | | | | | |
|  | GO:0002376 | Immune system process | 23 | 0.0343 | < 10^−5^ |
|  | GO:0006955 | Immune response | 16 | 0.0558 | < 10^−5^ |
|  | GO:0009607 | Response to biotic stimulus | 14 | 0.0342 | 0.0063 |
|  | GO:0043207 | Response to external biotic stimulus | 14 | 0.0352 | 0.0410 |
| KEGG | | | | | |
|  | gga05168 | Herpes simplex infection | 7 | 0.0583 | 0.0080 |
|  | gga04514 | Cell adhesion molecules | 6 | 0.0606 | 0.0121 |
